# Cuproptosis-related genes signature and validation of differential expression and the potential targeting drugs in temporal lobe epilepsy

**DOI:** 10.3389/fphar.2023.1033859

**Published:** 2023-06-26

**Authors:** Xiaolin Yang, Xiaoqing Zhang, Kaifeng Shen, Zhongke Wang, Guolong Liu, Kaixuan Huang, Zeng He, Yang Li, Zhi Hou, Shengqing Lv, Chunqing Zhang, Hui Yang, Shiyong Liu, Yanyan Ke

**Affiliations:** ^1^ Department of Neurosurgery, Second Affiliated Hospital, Army Medical University, Chongqing, China; ^2^ Department of Neurosurgery, Armed Police Hospital, Chongqing, China; ^3^ Guangyang Bay Laboratory, Chongqing Institute for Brain and Intelligence, Chongqing, China

**Keywords:** cuproptosis, Fdx1, LIPT1, temporal lobe epilepsy, diagnostic indicator, immune microenvironment

## Abstract

**Introduction:** Temporal lobe epilepsy (TLE) is the most common subtype of epilepsy in adults and is characterized by neuronal loss, gliosis, and sprouting mossy fibers in the hippocampus. But the mechanism underlying neuronal loss has not been fully elucidated. A new programmed cell death, cuproptosis, has recently been discovered; however, its role in TLE is not clear.

**Methods:** We first investigated the copper ion concentration in the hippocampus tissue. Then, using the Sample dataset and E-MTAB-3123 dataset, we analyzed the features of 12 cuproptosis-related genes in TLEs and controls using the bioinformatics tools. Then, the expression of the key cuproptosis genes were confirmed using real-time PCR and immunohistochemical staining (IHC). Finally, the Enrichr database was used to screen the small molecules and drugs targeting key cuproptosis genes in TLE.

**Results:** The Sample dataset displayed four differentially expressed cuproptosis-related genes (DECRGs; LIPT1, GLS, PDHA1, and CDKN2A) while the E-MTAB-3123 dataset revealed seven DECRGs (LIPT1, DLD, FDX1, GLS, PDHB, PDHA1, and DLAT). Remarkably, only LIPT1 was uniformly upregulated in both datasets. Additionally, these DECRGs are implicated in the TCA cycle and pyruvate metabolism—both crucial for cell cuproptosis—as well as various immune cell infiltrations, especially macrophages and T cells, in the TLE hippocampus. Interestingly, DECRGs were linked to most infiltrating immune cells during TLE’s acute phase, but this association considerably weakened in the latent phase. In the chronic phase, DECRGs were connected with several T-cell subclasses. Moreover, LIPT1, FDX1, DLD, and PDHB were related to TLE identification. PCR and IHC further confirmed LIPT1 and FDX1’s upregulation in TLE compared to controls. Finally, using the Enrichr database, we found that chlorzoxazone and piperlongumine inhibited cell cuproptosis by targeting LIPT1, FDX1, DLD, and PDHB.

**Conclusion:** Our findings suggest that cuproptosis is directly related to TLE. The signature of cuproptosis-related genes presents new clues for exploring the roles of neuronal death in TLE. Furthermore, LIPT1 and FDX1 appear as potential targets of neuronal cuproptosis for controlling TLE’s seizures and progression.

## Introduction

Epilepsy is a common and serious neurological disease worldwide, with temporal lobe epilepsy (TLE) being the most common subtype of epilepsy in adults. Furthermore, TLE is often associated with cognitive function impairment which is a common and disabling problem in individuals with TLE (Marissal, 2021). Moreover, the treatment of one-third of TLEs remains challenging because it shows a poor response to traditional chemotherapy (Marissal, 2021). Therefore, it is imperative to gain a deeper understanding of the molecular mechanisms of TLE to develop novel therapeutic strategies.

TLE is associated with anterior temporal epileptiform discharges, frequently accompanied by structural abnormalities in the hippocampal region, which are characterized by neuronal loss, gliosis, and sprouting mossy fibers (Blümcke et al., 2012). Recently, a study demonstrated that cuproptosis is a new cell death pattern (Tsvetkov et al., 2022), which could provide new hope for controlling neural loss in the hippocampus in TLE. Copper is an indispensable co-factor associated with various biological processes. The intracellular copper concentration influences human cell function through the mitochondrial TCA cycle and subsequent cuproptosis (Tsvetkov et al., 2022). Thus, dysregulation of copper homeostasis may trigger cytotoxicity, resulting in toxic protein stress and ultimately cell death (Tsvetkov et al., 2022). In addition, trace amounts of extra copper exacerbate hippocampal neurotoxicity and neuronal degeneration in cholesterol-fed mice (Lu et al., 2009). Intriguingly, studies in animals and humans have shown that copper levels in the epilepsy group are significantly elevated compared with those in healthy controls (Ristić et al., 2014; Vitale et al., 2019). In relation to this, copper deficiency in the brain also promotes neuron loss and the development of seizures in Menkes disease (Leiva and Infante, 2016). Furthermore, copper increases NMDA-driven calcium influx to induce neuronal hyperexcitability by releasing from synaptic vesicles after neuronal depolarization (Marchetti et al., 2014). In addition, prolonged copper exposure increases the enrichment of AMPA receptors in the neuronal plasma membrane to upregulate neuronal excitability (Scheiber et al., 2014). Copper also blocks GABAA and glycine receptors as neuromodulators to impair inhibitory synaptic transmission (Scheiber et al., 2014). Furthermore, some studies have demonstrated that copper significantly increases cerebral inflammatory factors, including nuclear factor kappa-light-chain-enhancer of activated B cells (NF-κB) p65, tumor necrosis factor α (TNF-α), and interleukin 6 (IL-6) (Sarawi et al., 2021), and inflammation has also been reported to increase copper levels in tissues (Hobin et al., 2022). Numerous studies have confirmed that immune cell infiltration and immune microenvironment contribute to the pathogenesis and progress of epilepsy (Yamanaka et al., 2021; Kumar et al., 2022). Therefore, we hypothesized that cuproptosis is involved in the mechanism of hippocampal neuronal death in TLE.

In this study, we identified and analyzed the key genes of cuproptosis involved in TLE using RNA-seq data of surgically removed hippocampus and autopsy samples and the public data in ArrayExpress through bioinformatics methods and confirmed them using real-time polymerase chain reaction (PCR) and immunohistochemical staining (IHC), wishing to find new targets for the diagnosis and drugs for the treatment of TLE and the targeting drugs.

## Materials and methods

### Clinical sample collection

Epileptic focus and control tissues were collected from the Department of Neurosurgery of Xinqiao Hospital. Informed consent was obtained from all patients. Finally, six epileptic focus specimens were obtained from the hippocampus of patients diagnosed with intractable TLE according to the International League Against Epilepsy (ILAE) diagnostic criteria (Blümcke et al., 2013). Because the hippocampal specimens from age-matched controls were sparse, we only collected two control hippocampi from autopsied individuals without a known history of neurologic or psychiatric disease, ensuring a 3:1 disease-to-control match. The clinical information of all samples is shown in Table 1. No statistical difference was observed in age or sex between TLE patients and controls. All study protocols were performed after obtaining permission from the Ethics Committee of the Army Medical University and in accordance with the ethical standards of the Declaration of Helsinki.

**TABLE 1:** The clinical information of TLE patients and controls.

Subjects	Gender	Age (years)	Course (years)	Preoperative AEDs	Seizure type	Frequency/Month	Resected tissue	Pathology	Application
1	F	25	8	TPM, CLB	FAS	64	LH	NL	RNA-seq, PCR, IHC
2	M	21	11	OXC, VPA	FIAS	90	LH	G, NL	RNA-seq, PCR, IHC
3	M	24	18	PHT, VPA	FIAS	35	RH	G, NL	RNA-seq, PCR, IHC
4	F	25	22	LTG, VPA, LEV	FBTCS	31	LH	G, NL	RNA-seq, PCR, IHC
5	M	20	9	VPA, PHT, PB	FIAS	118	RH	G, NL	RNA-seq, PCR, IHC
6	M	25	12	VPA, TPM, PB	FAS	16	RH	NL	RNA-seq, PCR, IHC
7	F	22	—	—	—	—	RH	normal	RNA-seq, PCR, IHC
8	M	32	—	—	—	—	LR	normal	RNA-seq, PCR, IHC
9	M	21	—	—	—	—	RH	normal	PCR, IHC

F, female; M, man; TPM, topiramate; CLB, clonazepam; OXC, oxcarbazepine; VPA, valproic acid; PHT, phenytoin; LTG, lamotrigine; LEV, levetiracetam; PB, phenobarbital; FIAS, focal impaired awareness seizure; FAS, focal aware seizure; FBTCS, focal-to-bilateral tonic-clonic seizure. RH, right hippocampus; LR, left hippocampus; G, gliosis; NL, neuron loss. RNA-seq, RNA, sequencing; PCR, polymerase chain reaction; IHC, immunohistochemistry.

### Detection of tissue copper ion concentration

Tissue copper colorimetric assay kit was using to detected the copper concentration of the hippocampus tissue. Briefly, the sample tissue of about 500 mg was obtained and weighed weight, and immediately put into a liquid nitrogen overnight. The next day, precooled reagent B was added and put into homogenizer and homogenize, then centrifuge at 4°C for 5 min at 16000 g. Carefully take 500 μL of supernatant into the new tube and use 10 μL solution for protein quantification. Add anti-interference solution (Reagent C), oscillate for 10 s and centrifuge at 4°C for 10 min at 16000 g. Carefully take the supernatant into the new tube and immediately place on the ice. Before detection, Reagent D was added to Reagent E to obtain working solution and take 200 μL to the sample solution, incubate at 37°C for 5 min, away from light. Then put into the spectrophotometer for detection to get the optical density (OD) and calculate the copper concentration of the sample.

### RNA sequencing

All collected samples were immediately placed in centrifuge tubes containing TRIzol and stored at −20 °C until total RNA extraction. Total RNA was isolated from the sample homogenate, according to the manufacturer’s instructions (Invitrogen, United States). Next, the quantity and quality of total RNA were assessed using a Nanodrop spectrophotometer (NanoDrop Technologies, United States). RNA samples were included in the subsequent study with an A260/A280 ratio above 1.80 and an A260/A230 ratio above 2.00. RNA integrity was further evaluated using the Agilent 2,200 TapeStation (Agilent Technologies, United States), and samples with an RNA Integrity Number under 7.0 was excluded and the total RNA was re-extracted. Ribosomal RNA was removed using the Epicentre Ribo-Zero rRNA Removal Kit (Illumina, United States), and RNA was broken into fragments of approximately 200 bp. The fragmented RNAs were then used to synthesize first- and second-strand cDNA, and adaptor ligation and enrichment with a low-cycle were performed as instructed by the TruSeq^®^ RNA LT/HT Sample Prep Kit (Illumina, United States). Qubit^®^2.0 (Life Technologies, United States) and Agilent 2,200 TapeStation were used to evaluate the purified library products. Next, they were diluted to 10 pM for *in situ* cluster generation on a HiSeq2500 pair-end flow cell. Following quality control of raw sequencing, the data were aligned to the human GRCh38 genome using TopHat (version 1.0.12). And then, we get the data from human hippocampus tissue samples (Sample dataset).

### Identification of differentially expressed genes

Estimation of transcription abundance in each gene was determined using Cufflinks (v1.0.3) in collected samples. A gene transfer format file for reference genome annotation was obtained from the UCSC database. After filtering out non-expressed or low-expressed genes (at least one count/million), the data were normalized. These data were referred as the sample data. Then, differentially expressed cuproptosis-related genes (DECRGs) were identified between TLEs and controls in the sample data using the DESeq2 R package and ArrayExpress data using the Limma R package. Genes with a fold-change (FC) > 1 and adjust *p*-value< 0.05 were considered differentially expressed genes between TLEs and controls, and the DECRGs were obtained using Venny 2.1 (https://bioinfogp.cnb.csic.es/tools/venny).

### Real-time PCR experiments

Total RNA was isolated from each human sample using 1 mL RNAiso Plus reagent (TaKaRa, Otsu, Japan), as described previously (Zhang et al., 2022). RNA concentration and quality were estimated at 260/280 nm using an Ocean Optics spectrophotometer (Dunedin, United States). RNA was reverse-transcribed into cDNA using oligo (dT) primers. Next, the PCR mixture consisting of TB Green (10 uL), forward and reverse primers (10 uL, TAKARA, Japan), DNA (1 uL) and ddH2O (7.0 uL) were placed in the Realtime System (CFX Connect, United States). The reaction parameters were 95°C for 5 min (1 cycle), followed by 40 cycles of 95°C for 30°s, and 60°C for 30 s. The relative expression of mRNA was quantified using the 2^−ΔΔCT^ method.

### Immunohistochemical staining

Briefly, the graded alcohol series was used to deparaffinize and dehydrate paraffin-embedded human brain sections. Endogenous peroxidase activity was quenched by 3% H_2_O_2_ for 30 min on paraffin sections which then boiled for 30 min in antigen retrieval buffer (pH6.0 citrate buffer). The 5% bovine serum albumin solution and 0.3% Triton solution were then applied for 1 h at room temperature to block the sections. After the sections were washed three times with PBS, all sections were incubated with primary rabbit anti-human LIPT1 (1:100, Bioss, China) and FDX1 (1:100, Bioss, China) overnight at 4°C. The HRP-conjugated goat anti-rabbit antibody (Boster, China) was applied to the sections for 1 h at 37°C after several washing (4-5 times) with PBS. Then, the immunoreactivity was visualized using 3, 3-diaminobenzidine (DAB) substrate solution (Boster, China). Finally, sections were cleared in xylene after alcohol dehydration, and then coverslipped. The sections incubated with primary antibody free solution, tenfold excess specific blocking antigen and isotype-matched rabbit polyclonal antibody were used as a negative control, and did not exhibit immunoreactive cells. All sections were viewed and capture the pictures under an Olympus BX63 microscope (Olympus, Tokyo, Japan). All pictures were taken with the same aperture, power, and exposure time.

### Array express data acquisition

To validate the results of our sample, the Array Express database (https://www.ebi.ac.uk/biostudies/arrayexpress) was used to study gene expression in human TLE. The inclusion criteria were as follows: temporal lobe epilepsy, organism: *Homo sapiens*, experiment type: RNA assay and Array assay, and samples including the hippocampus. Based on the above conditions, a comprehensive search of the ArrayExpress databases was conducted from inception to 10 April 2023 resulting in only one ArrayExpress dataset, E-MTAB-3123. In the study. we also use the dataset of animal model of TLE (including the dataset of acute, latent and chronic stage of TLE) to explore the impact of this correlation between DECRGs and immune cells infiltration on the occurrence and progression of TLE, including GSE49030, GSE88992, GSE49849 and GSE14763. Data were processed and normalized using the Limma package in the R environment.

### Expression of key cuproptosis-related genes

Based on previous studies (Bian et al., 2022; Tsvetkov et al., 2022), 12 key cuproptosis-related genes were selected, including CDKN2A, FDX1, DLD, DLAT, LIAS, GLS, LIPT1, MTF1, PDHA1, PDHB, ATP7B, and SLC31A1. The expression of these cuproptosis-related genes was analyzed in the Sample dataset and E-MTAB-3123 dataset to obtain DECRGs between TLE and control patients.

### Construction of protein–protein interaction network and community detection

To explore the potential protein–protein interactions (PPI) associated with TLE, the list of DECRGs was mapped on the STRING online database (medium confidence score: 0.4, maximum number of interactors to show on shell: ≤10). Then, genes interacting with DECRGs were utilized to analyze the related GO function and KEGG pathways. Communities in the PPI network were detected and visualized using the Cytoscape (v3.7). To further explore the interaction pairs associated with TLE, MCODE, a Cytoscape plugin, was used to screen the cuproptosis-related modules in the PPI network. The parameters of the MCODE plugin were as follows: degree cutoff = 2, node score cutoff = 0.2, and K-score = 2. Modules with scores >3 and nodes >6 were identified as significant.

### Analysis of immune infiltration by CIBERSORTx and ssGSEA

To evaluate the relative abundance of immune cells infiltrating the hippocampus in TLE, the CIBERSORTx algorithm (https://cibersort.stanford.edu/) was utilized to transform the gene expression matrix into the composition of immune cells. We obtained a CIBERSORTx output with a *p*-value >0.05, to accurately evaluate the filtrated abundance of immune cell composition. In addition, the ssGSEA, algorithm using R package “GSVA” was introduced to validate the relative infiltration abundance of immune cell types. To obtain the specific immune cell subtypes in epileptic foci, a two-tailed Wilcoxon test was performed to evaluate the differences in immunoscores between the hippocampal tissues of control and TLE patients.

### Analysis of correlation between immune infiltration and DECRGs

The relative abundance of infiltrated immune cells was used to detect the relationship between the expression of DECRGs and the abundance of 22 immune cells (including T cells, B cells, plasma cells, natural killer cells, monocytes, mast cells, eosinophils, neutrophils, dendritic cells, and macrophages) using the CIBERSORTx algorithm and 28 immune cells (including T cells, B cells, dendritic cells, natural killer cells, eosinophils, macrophages, mast cells, MDSC, monocytes, and neutrophils) using the ssGSEA algorithm. Spearman correlation analysis was used to detect the association between the abundance of immune cells and DECRGs, *p* < 0.05 was regarded as a significant correlation.

### Receiver operating characteristic curves of the five key genes

To assess the diagnostic values of the DECRGs for TLE, the accuracy of each gene in identifying TLE was evaluated using the area under the curve (AUC) value of the receiver operating characteristic (ROC) curve. We conducted ROC analysis using the pROC package to verify the classification efficacy of DECRGs in the E-MTAB-3123 dataset and validated it using the Sample dataset.

### Prediction of small molecules or drugs which target key cuproptosis-related genes

To explore drugs targeting the key cuproptosis-related genes in TLE, we analyzed small molecules and drugs which targeted the key cuproptosis-related genes using the Enrichr database (https://maayanlab.cloud/Enrichr). The drug with the highest combined score and adjusted *p*-value <0.05 in a single gene or multiple genes was considered as the drug targeting the key cuproptosis-related gene.

### Statistical analysis

R software was used to perform statistical analyses (R version 4.1.3). Differences were considered statistically significant at a two-sided *p*-value of <0.05.

## Results

### Copper ion concentration and ceruloplasmin expression in the hippocampus of TLEs and controls

A recent study shows that copper ion concentration may involve neuronal cuproptosis (Tsvetkov et al., 2022). Therefore, we first observed the copper ion concentration and ceruloplasmin expression in the hippocampus tissues of TLEs and controls. The results showed that the concentration of copper increased in the hippocampus tissue of TLEs compared to controls, but no statistical difference (Supplementary Figure S1A, controls vs. TLEs *p* = 0.0547). And there was no significant difference of ceruloplasmin expression between TLEs and controls in Sample dataset (controls vs. TLEs *p* = 0.3736) and E-MTAB-3123 dataset (controls vs. TLEs *p* = 0.5575) (Supplementary Figures S1B, C). So, the cuproptosis maybe depend on not only the concentration of copper but also the cuproptosis genes.

### Identification and validation of DECRGs

**FIGURE 1:**
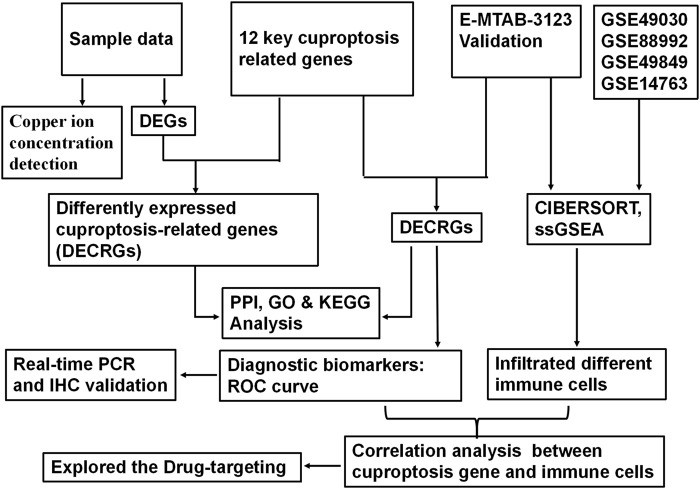
Schematic diagram illustrating the flow of analysis in the study.

**FIGURE 2:**
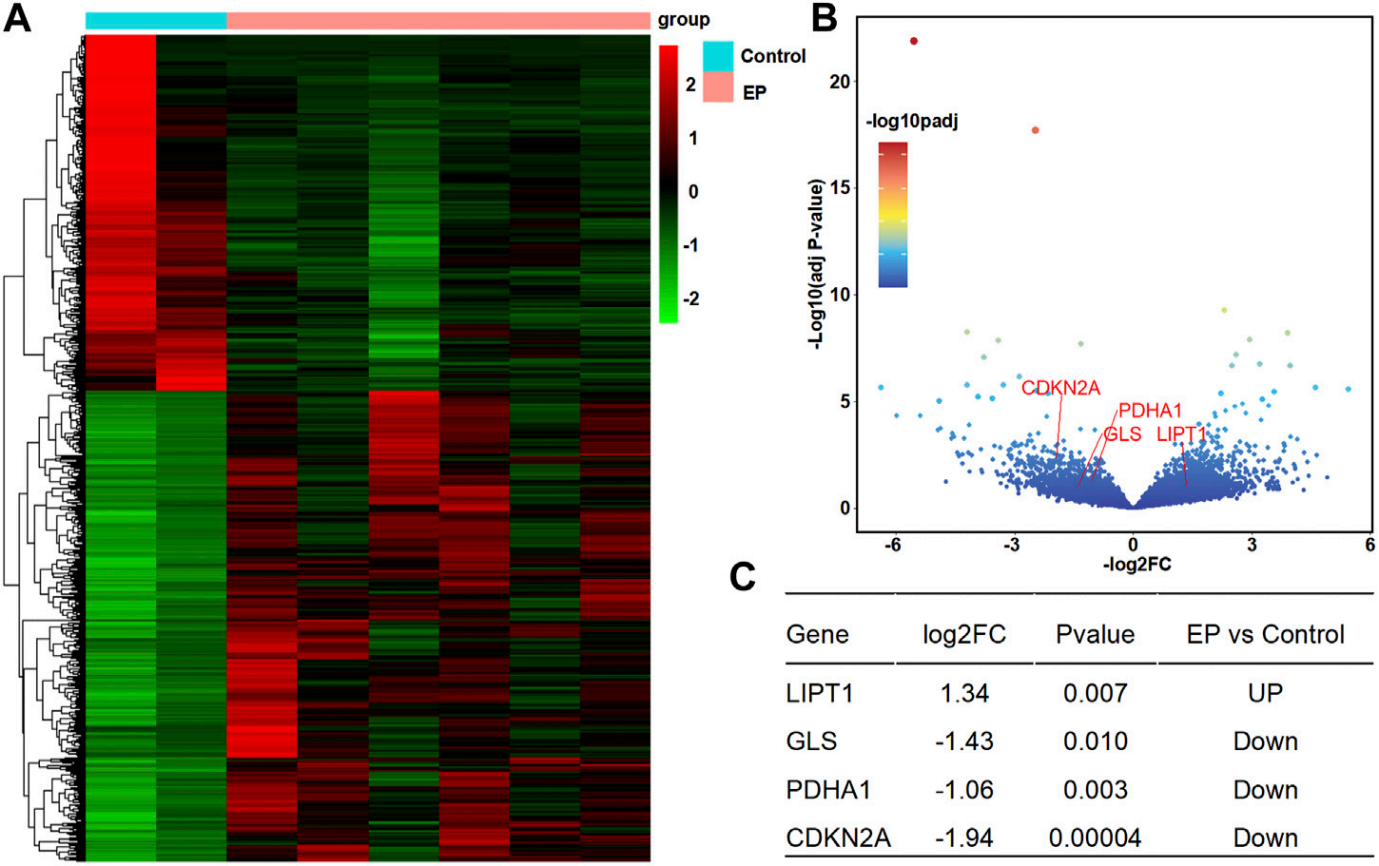
Differentially expressed cuproptosis-related genes (DECRGs) analysis in the resected sample data. **(A)** Heatmap of differentially expressed genes; **(B)** Volcano plot of gene expression, red marker is the DECRGs; **(C)** Differentially expressed cuproptosis-related genes.

**FIGURE 3:**
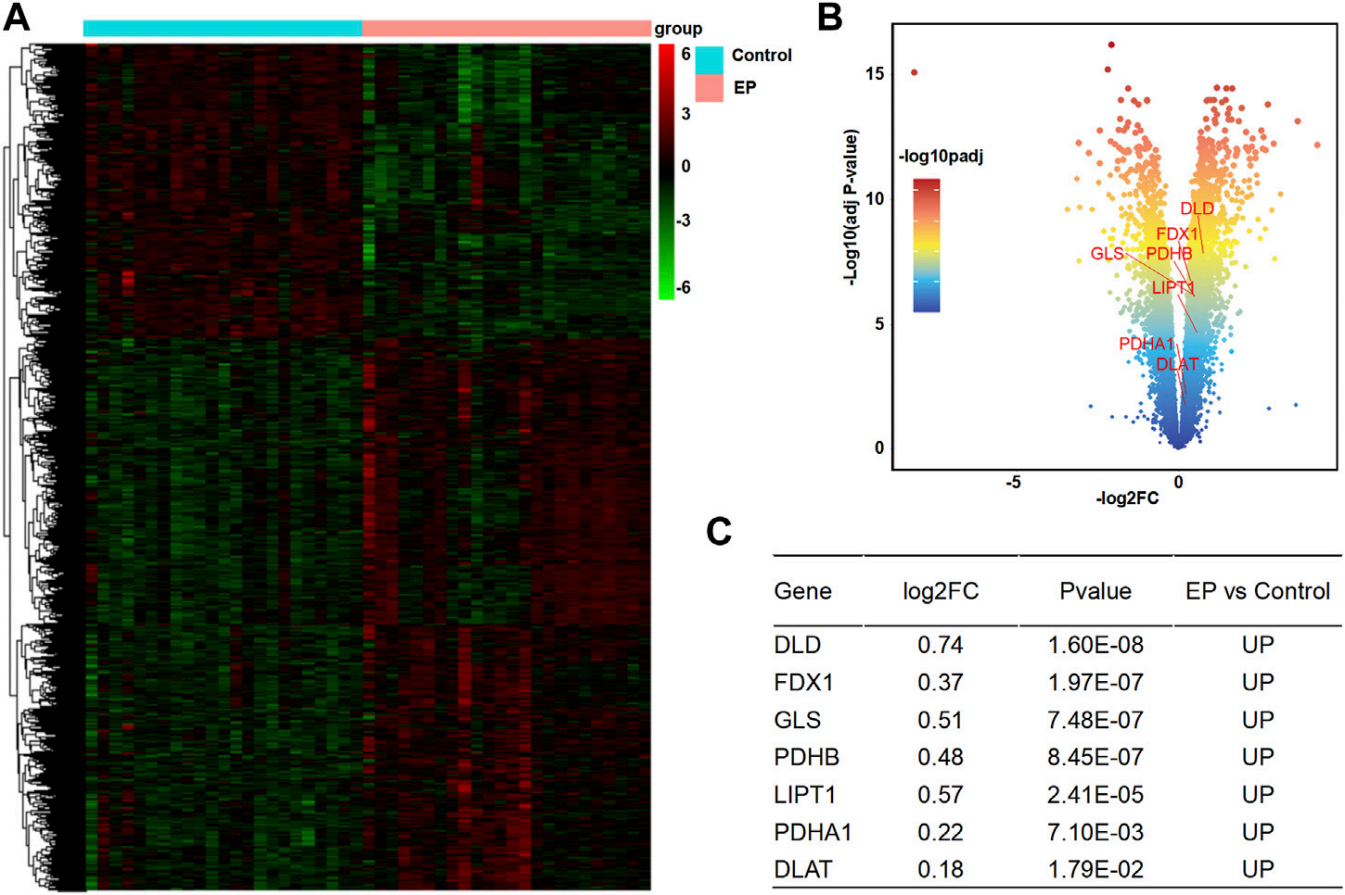
Analysis of DECRGs in the E-MTAB-3123 data. **(A)** Heatmap of differentially expressed genes; **(B)** Volcano plot of gene expression, red marker is the DECRGs; **(C)** Differentially expressed cuproptosis-related genes.

According to the analysis scheme (Figure 1), we first identified DECRGs between TLE and controls in the sample data. There were four significantly different genes between control and epilepsy hippocampus samples, of which LIPT1 (log2 (fold change) = 1.34, *p* = 7.04 × 10^−3^) showed significantly higher expression while GLS (log2 (fold change) = −1.43, *p* = 1.03 × 10^−2^), PDHA1 (log2 (fold change) = −1.06, *p* = 3.01 × 10^−3^), and CDKN2A (log2 (fold change) = −1.94, *p* = 4.38 × 10^−5^) showed lower expression in TLE than controls (Figures 2A–C). Moreover, in the validation data E-MTAB-3123, seven genes had significantly higher expression in TLE than controls, which consisted of DLD (log2 (fold change) = 0.74, *p* = 1.6 × 10^−8^), FDX1 (log2 (fold change) = 0.37, *p* = 1.97 × 10^−7^), PDHB (log2 (fold change) = 0.48, *p* = 8.45 × 10^−7^), LIPT1 (log2 (fold change) = 0.57, *p* = 2.41 × 10^−5^), GLS (log2 (fold change) = 0.51, *p* = 7.48 × 10^−7^), PDHA1 (log2 (fold change) = 0.22, *p* = 7.10 × 10^−3^), and DLAT (log2 (fold change) = 0.18, *p* = 1.8 × 10^−2^) (Figures 3A–C). Interestingly, only LIPT1 was upregulated in both the sample data and E-MTAB-3123 data. The results indicated that cuproptosis could be involved in TLE; however, the function and interaction of DECRGs are unclear. Therefore, according to the previous study (Tsvetkov et al., 2022), we included all the different expression cuproptosis genes into the subsequent analysis.

### PPI network of DECRGs and functional enrichment analysis

**FIGURE 4:**
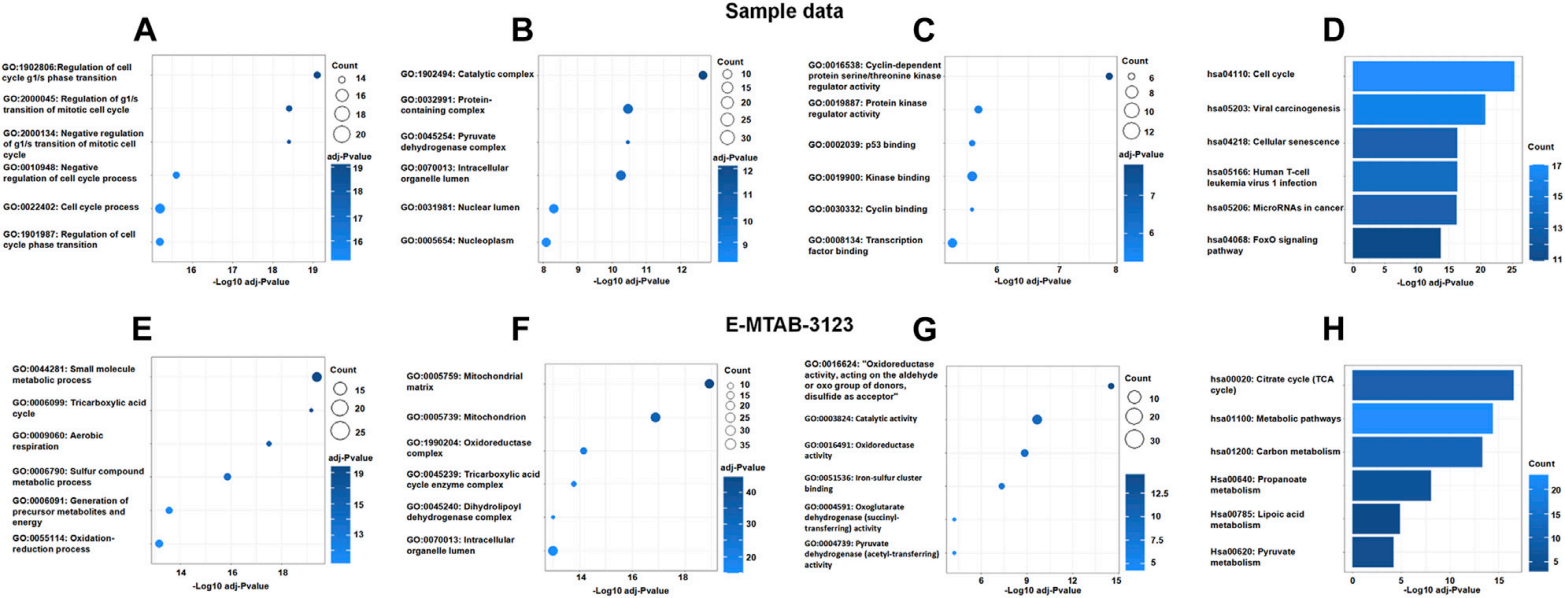
The Go enrichment and KEGG pathway enrichment of DECRGs related genes. **(A,E)** Biological process analysis of DECRGs related genes; **(B,F)** Cellular component analysis of DECRGs related genes; **(C,G)** Molecular function analysis of DECRGs related genes; **(D,H)** The KEGG pathway enrichment analysis of DECRGs related genes.

To explore the interaction between DECRGs, and analyze the roles of DECRGs related genes, PPI analyses were performed using the STRING web. The results showed there are close interactions between DECRGs in the Sample data and E-MTAB-3123 data. For the Sample data, the Gene Ontology results showed that the most significant enrichment terms of DECRGs were mainly involved in the regulation of the cell cycle (biological process); the cellular components were located in the catalytic complex, protein-containing complex, pyruvate dehydrogenase complex, intracellular organelle lumen, nuclear lumen, and nucleoplasm; the molecular function included the cyclin-dependent protein serine/threonine kinase regulator activity, protein kinase regulator activity, p53 binding, kinase binding, cyclin binding, and transcription factor binding (Figures 4A–C); the results of KEGG enrichment analysis showed that the most important pathway was the following: cell cycle, viral carcinogenesis, cellular senescence, human T-cell leukemia virus 1 infection, microRNAs in cancer, and FoxO signaling pathway (Figure 4D). For the E-MTAB-3123 data, the Gene Ontology results showed that the most significant enrichment terms of DECRGs were mainly involved in mitochondrial metabolism, and the results of KEGG enrichment analysis showed that the DECRGs were involved in the TCA cycle (Figures 4E–H).

### Subset analysis results of PPI network

**FIGURE 5:**
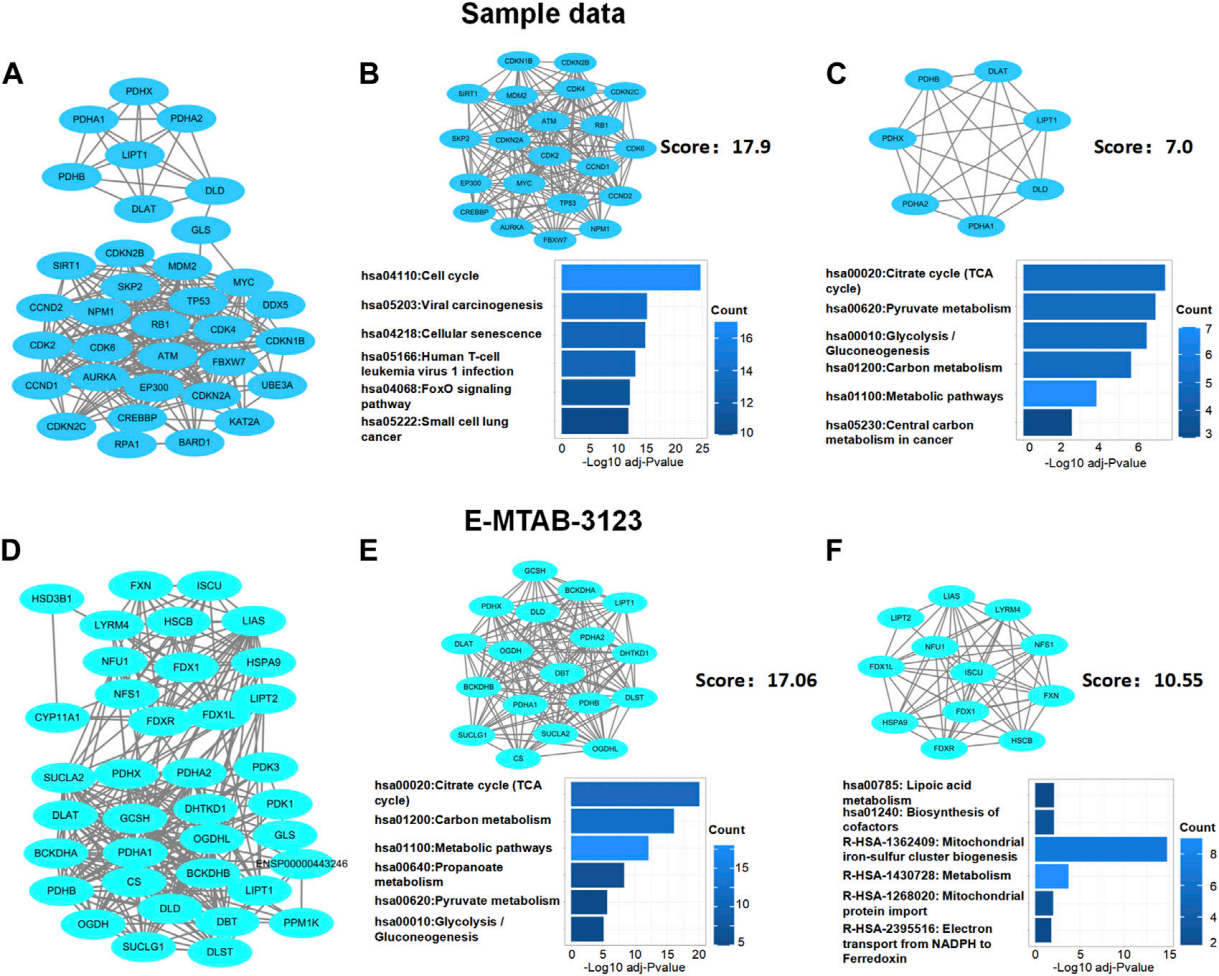
Protein–protein interaction network analysis of DECRGs and DECRGs related genes. **(A,D)** Protein–protein interaction network of DECRGs and DECRGs related genes; **(B,C,E,F)** Module analysis for DECRGs related genes and KEGG enrichment analysis for genes in module.

Next, based on network module analysis using the MCODE plugin in Cytoscape (Figure 5), the network of sample data clustered into two modules: cluster 1, which had 21 genes and 179 edges, with a score of 17.9 which mainly responded to the cell cycle KEGG pathway; cluster 2, which had seven genes and 21 edges with the TCA cycle KEGG pathway (Figures 5A–C). In the E-MTAB-3123 data, there were also two main clusters: cluster 1, which had 18 genes, 145 edges, and a score of 17.06, with the TCA cycle KEGG pathway; 12 genes and 58 edges in cluster 2 of the network were mainly responsible for the mitochondrial metabolic process (Figures 5D–F). These results indicated that cuproptosis, which is dependent on the mitochondrial TCA cycle, could be involved in TLE.

### Immune cells infiltration in TLE

**FIGURE 6:**
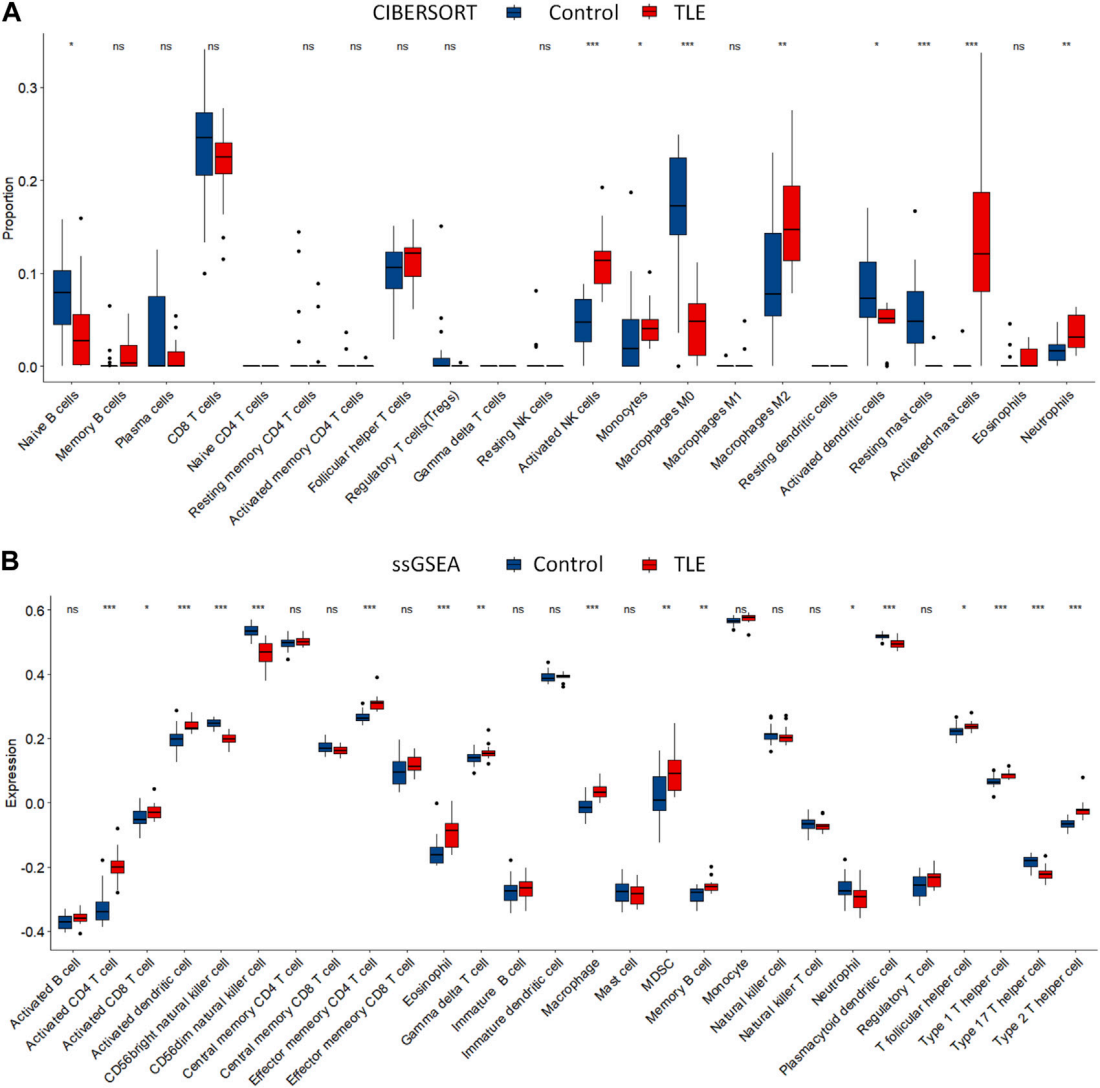
Immune cells infiltration analysis. The abundance of immune cell in the hippocampus of TLE of **(A)** CIBERSORT algorithm and **(B)** ssGSEA algorithm.

Extensive studies demonstrate that immune cells infiltration contribute to epileptogenesis and progress of epilepsy (Wang et al., 2014; Yamanaka et al., 2021; Kumar et al., 2022). In addition, copper significantly increases the levels of cerebral inflammatory factors (Sarawi et al., 2021). Therefore, we analyzed immune cell infiltration in TLE and controls in E-MTAB-3123 data using the CIBERSORTx and ssGSEA algorithms. The results of CIBERSORTx analysis showed that among 22 types of immune cells, activated NK cells (*p* < 0.001), M2 macrophages (*p* < 0.05), activated mast cells (*p* < 0.001), and neutrophils (*p* < 0.001) were more abundant in the hippocampal tissues of TLE than in controls (Figure 6A). Regulatory T cells (Tregs) (*p* < 0.05), M0 macrophages (*p* < 0.001), activated dendritic cells (*p* < 0.05), and resting mast cells (*p* < 0.001) significantly decreased in TLE hippocampal samples compared to controls. In addition, we adopted another immune cell-type quantification method, ssGSEA, to quantify the enrichment scores of immune cell types. The scores of activated CD4 T cells (*p* < 0.001), effector memory CD4 T cells (*p* < 0.001), macrophages (*p* < 0.001), activated CD8 T cells (*p* < 0.05), activated dendritic cells (*p* < 0.001), eosinophils (*p* < 0.001), gamma delta T cells (*p* < 0.01), MDSC (*p* < 0.01), memory B cells (*p* < 0.01), T follicular helper cells (*p* < 0.05), type 1 T helper cells (*p* < 0.001), and type 2 T helper cells (*p* < 0.001) increased in the hippocampal samples of TLE compared to the controls, while the scores of CD56 bright NK cells (*p* < 0.001), CD56 dim NK cells (*p* < 0.001), neutrophils (*p* < 0.05), plasmacytoid dendritic cells (*p* < 0.001), and type 17 T helper cells (*p* < 0.001) decreased. Based on the above results, macrophages and T cells might be the key immune-infiltrating cells in the hippocampus in TLE.

### Correlation between DECRGs and immune cells infiltration

**FIGURE 7:**
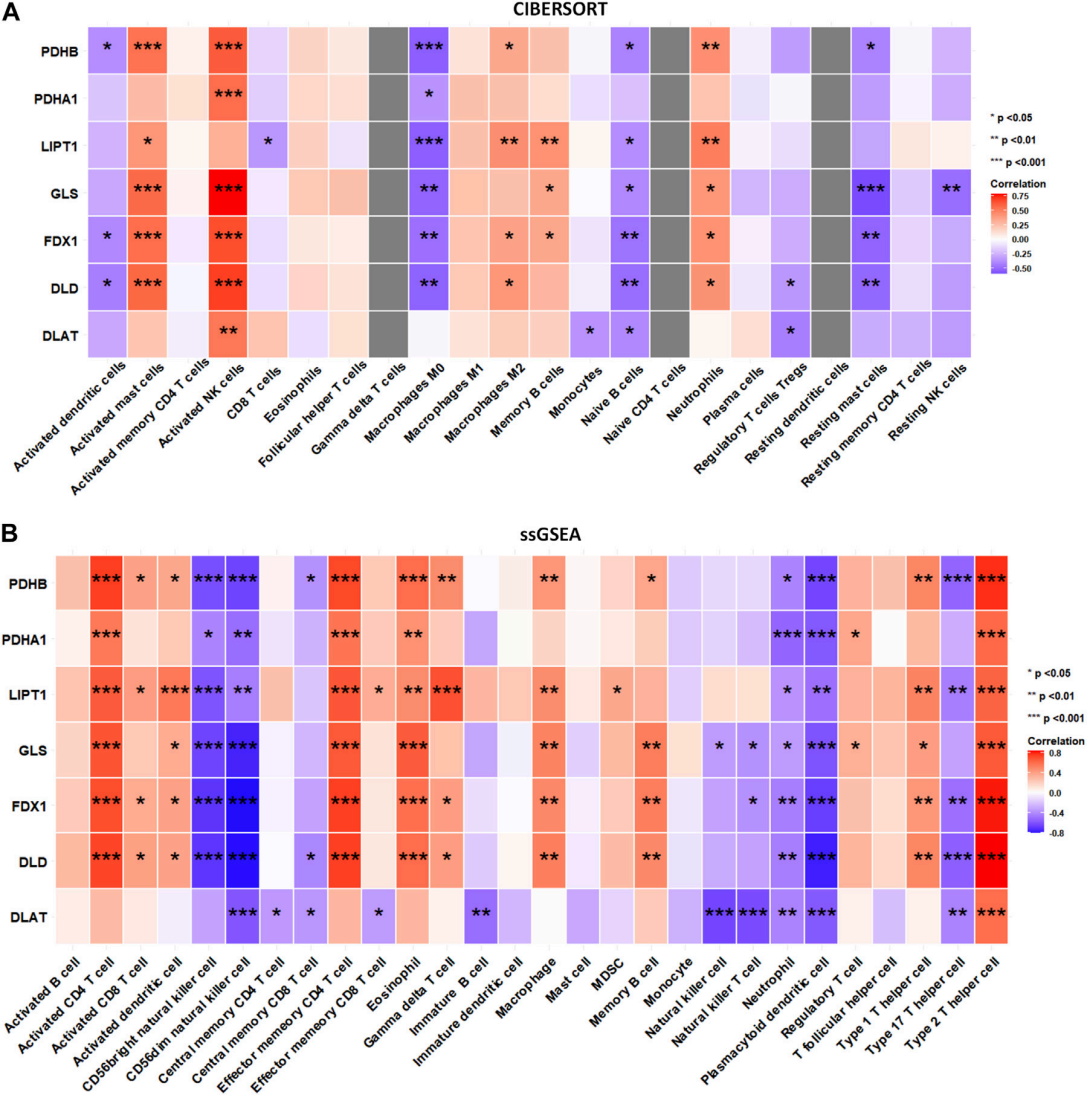
The relation between infiltrated immune cells and differentially expressed cuproptosis relative genes (DECRGs). **(A)** The relation between DECRGs and the score of infiltrated immune cells of CIBERSORT algorithm; **(B)** The relation between DECRGs and the score of infiltrated immune cells of ssGSEA algorithm.

To explore whether DECRGs were related to immune cell infiltration in TLE, Spearman’s correlation analysis was performed using the E-MTAB-3123 data. The DECRGs were significantly related to CIBERSORT scores of dendritic cells, M0 macrophages, NK cells, mast cells, neutrophils, and CD4 T cells (Figure 7A). The DECRGs were also significantly related to the ssGSEA scores of CD4 T cells, CD8 T cells, dendritic cells, NK cells, eosinophils, gamma delta T cells, macrophages, B cells, neutrophils, and T helper cells (Figure 7B). Based on these results and previous studies (Silverberg et al., 2010; Ouédraogo et al., 2021; Kumar et al., 2022), we found that DECRGs are closely related to macrophages and T cells. FDX1 (R = −0.49, *p* = 0.0031), LIPT1 (R = −0.48, *p* = 0.0037), DLD (R = 0.52, *p* = 0.0014), PDHB (R = −0.53, *p* = 0.001), and GLS (R = −0.53, *p* = 0.0011) significantly negatively correlated with M0 macrophages, whereas LIPT1 (R = 0.39, *p* = 0.021) and DLD (R = 0.37, *p* = 0.030) significantly positively correlated with M2 macrophages (Figure 7A). FDX1 (R = 0.70, *p* = 3.40 × 10^−6^), LIPT1 (R = 0.65, *p* = 2.02 × 10^−5^), DLD (R = 0.71, *p* = 1.51 × 10^−6^), PDHA1 (R = 0.55, *p* = 6.8 × 10^−4^), PDHB (R = 0.74, *p* = 3.99 × 10^−7^), and GLS (R = 0.67, *p* = 1.04 × 10^−5^) significantly positively correlated with activated CD4 T cells (Figure 7B); moreover, FDX1 (R = 0.73, *p* = 3.40 × 10^−7^), LIPT1 (R = 0.66, *p* = 1.37 × 10^−5^), DLD (R = 0.73, *p* = 5.87 × 10^−7^), PDHA1 (R = 0.56, *p* = 4.23 × 10^−4^), PDHB (R = 0.71, *p* = 1.85 × 10^−6^), and GLS (R = 0.66, *p* = 1.62 × 10^−5^) also significantly positively correlated with the abundance of effector memory CD4 T cells (Figure 7B). Furthermore, FDX1 (R = 0.43, *p* = 9.41 × 10^−3^), LIPT1 (R = 0.51, *p* = 1.90 × 10^−3^), DLD (R = 0.50, *p* = 2.15 × 10^−3^), PDHB (R = 0.50, *p* = 2.14 × 10^−3^), and GLS (R = 0.40, *p* = 1.68 × 10^−2^) also significantly positively correlated with the abundance of Th1 cells, and FDX1 (R = −0.50, *p* = 1.99 × 10^−3^), LIPT1 (R = −0.45, *p* = 6.55 × 10^−3^), DLD (R = −0.56, *p* = 4.87 × 10^−4^), DLAT (R = −0.44, *p* = 8.63 × 10^−3^), and PDHB (R = −0.54, *p* = 7.37 × 10^−4^) also significantly negatively correlated with the abundance of Th17 cells (Figure 7B).

### Correlation between DECRGs and immune cells infiltration in the occurrence and progression of TLE

To perform an in-depth analysis of the impact of this correlation between DECRGs and immune cell infiltration on the occurrence and progression of TLE, we used the data from animal model of temporal lobe epilepsy (including the data of acute, latent, and chronic stages of TLE).

**FIGURE 8:**
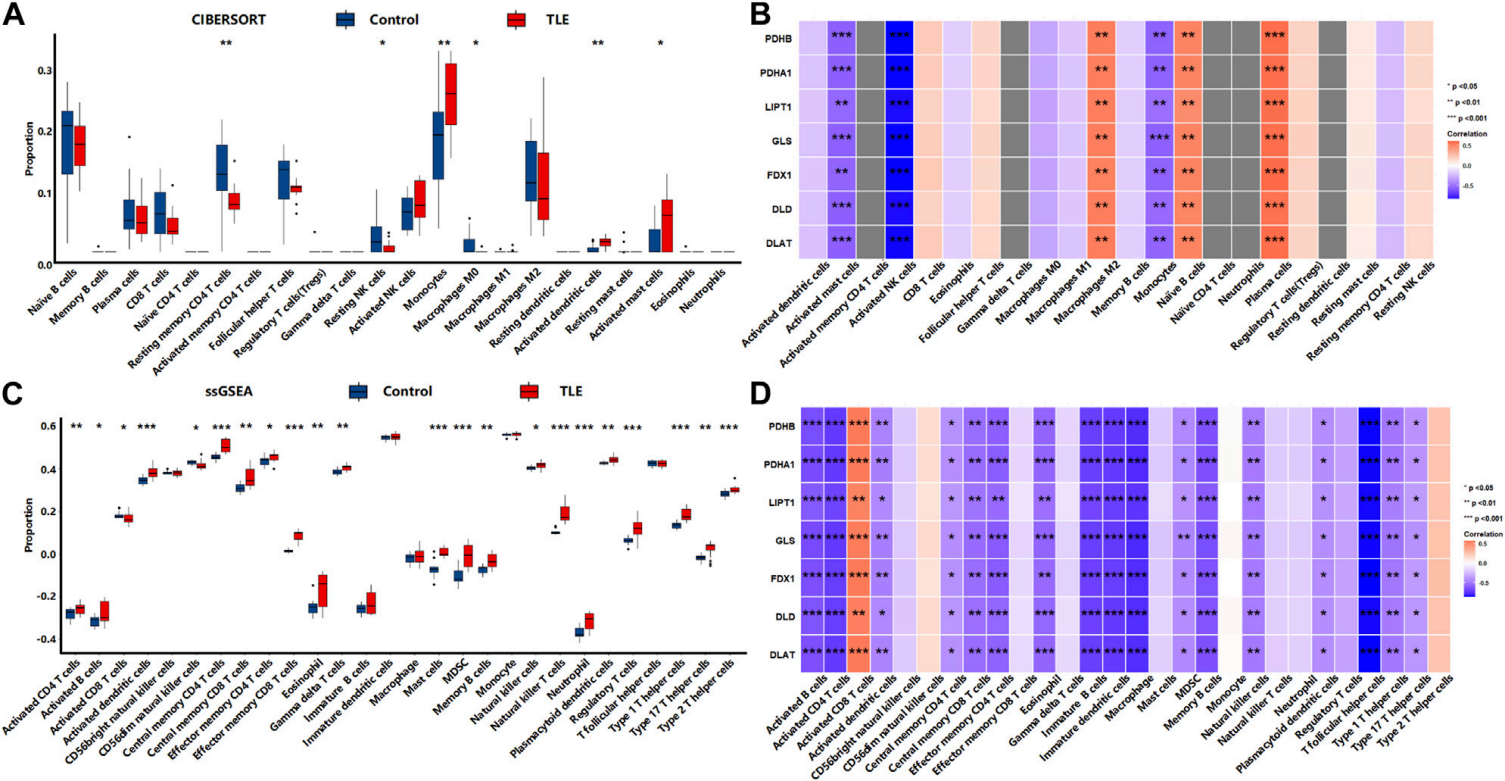
The immune cells infiltration analysis in the acute stage of TLE. **(A,B)** The abundant of immune cell in the hippocampus of TLE of CIBERSORT algorithm and the correlation with DECRGs. **(C,D)** The abundant of immune cell in the hippocampus of TLE of ssGSEA algorithm and the correlation with DECRGs.

The results showed that in the acute stage, CIBERSORTx analysis (Figure 8A) showed that among 22 types of immune cells, Monocytes (*p* < 0.01), Activated dendritic cells (*p* < 0.01), and Activated mast cells (*p* < 0.05) were more abundant in the hippocampal tissues of TLE than in controls; Resting memory CD4 T cells (*p* < 0.01), Resting NK cells (*p* < 0.05) and Macrophages M0 (*p* < 0.05) significantly decreased in TLE hippocampal samples compared to controls. Correlation analysis (Figure 8B) showed DECRGs (PDHB, PDHA1, LIPT1, GLS, FDX1, DLD, and DLAT) were related to the abundance of Macrophages M2, Activated mast cells, Activated NK cells, Monocytes, Naïve B cells, and plasma cells. The ssGSEA analysis (Figure 8C) showed that among 28 types of immune cells, Activated CD4 T cells (*p* < 0.01), Activated B cells (*p* < 0.05), Activated dendritic cells (*p* < 0.001), Central memory CD4 T cells (*p* < 0.001), Central memory CD8 T cells (*p* < 0.01), Effector memory CD4 T cells (*p* < 0.05), Effector memory CD8 T cells (*p* < 0.001), Eosinophil (*p* < 0.01), Gamma delta T cells (*p* < 0.01), Mast cells (*p* < 0.001), MDSC (*p* < 0.001), Memory B cells (*p* < 0.01), Natural killer cells (*p* < 0.05), Natural killer T cells (*p* < 0.001), Neutrophil (*p* < 0.001), Plasmacytoid dendritic cells (*p* < 0.01), Regulatory T cells (*p* < 0.001), Type 1 T helper cells (*p* < 0.001), Type 2 T helper cells (*p* < 0.01) and Type 17 T helper cells (*p* < 0.001) were more abundant in the hippocampal tissues of TLE than in controls. Activated CD8 T cells (*p* < 0.05) and CD56dim natural killer cells (*p* < 0.05) significantly decreased in TLE hippocampal samples compared to controls. Correlation analysis (Figure 8D) showed all DECRGs were closely related to the abundance of Activated CD4 T cells, Activated B cells, Activated dendritic cells, Central memory CD4 T cells, Central memory CD4 T cells, Central memory CD8 T cells, Effector memory CD4 T cells, Eosinophil, Immature B cells, Immature dendritic cells, Macrophage, MDSC, Memory B cells, Natural killer cells, Plasmacytoid dendritic cells, T follicular helper cells, Type 1 T helper cells, and Type 17 T helper cells.

**FIGURE 9:**
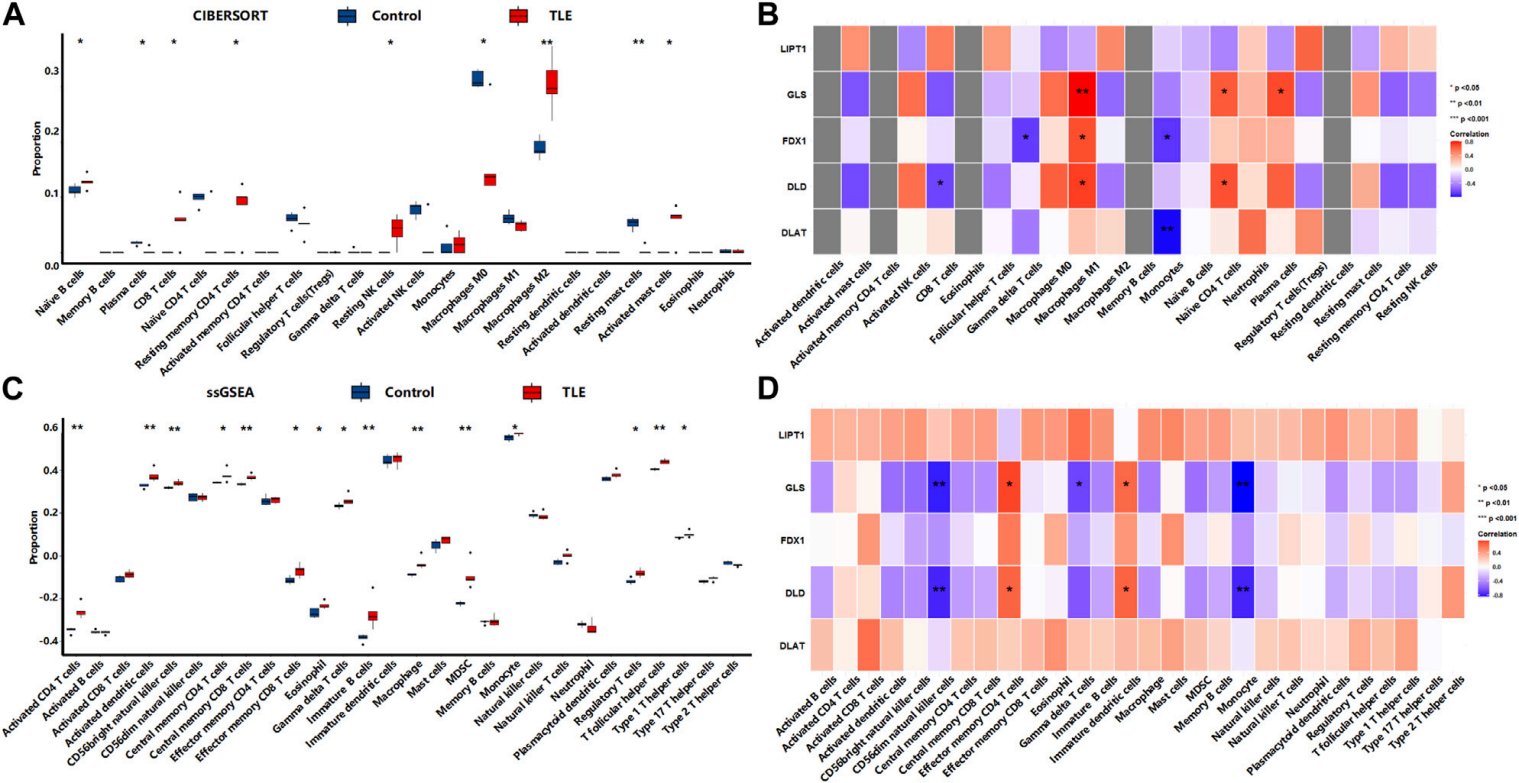
The immune cells infiltration analysis in the latent stage of TLE. **(A,B)** The abundant of immune cell in the hippocampus of TLE of CIBERSORT algorithm and the correlation with DECRGs. **(C,D)** The abundant of immune cell in the hippocampus of TLE of ssGSEA algorithm and the correlation with DECRGs.

In the latent stage, CIBERSORTx analysis (Figure 9A) showed that among 22 types of immune cells, Naïve B cells (*p* < 0.05), CD8 T cells (*p* < 0.05), Resting memory CD4 T cells (*p* < 0.05), Resting NK cells (*p* < 0.05), Macrophages M2 (*p* < 0.01), and Activated mast cells (*p* < 0.05) were more abundant in the hippocampal tissues of TLE than in controls (Figure 6A). Plasma cells (*p* < 0.01), Macrophages M0 (*p* < 0.05), and Resting mast cells (*p* < 0.01) significantly decreased in TLE hippocampal samples compared to controls. Correlation analysis (Figure 9B) showed GLS was related to the abundance of Macrophages M1, Naïve CD4 T cells, and plasma cells; FDX1 was closely related to the abundance of Gamma delta T cells, Macrophages M1, and Monocytes. DLD was related to the abundance of CD8 T cells, Macrophages M1, and Naïve CD4 T cells. DLAT was closely related to the abundance of Monocytes. The ssGSEA analysis (Figure 9C) showed that among 28 types of immune cells, Activated CD4 T cells (*p* < 0.01), Activated dendritic cells (*p* < 0.01), CD56bright natural killer cells (*p* < 0.01), Central memory CD4 T cells (*p* < 0.05), Central memory CD8 T cells (*p* < 0.01), Effector memory CD8 T cells (*p* < 0.05), Eosinophil (*p* < 0.05), Gamma delta T cells (*p* < 0.05), immature B cells (*p* < 0.01), Macrophage (*p* < 0.01), MDSC(*p* < 0.01), Monocyte (*p* < 0.05), Regulatory T cells (*p* < 0.05), T follicular helper cells (*p* < 0.01), and Type 1 T helper cells (*p* < 0.05) were more abundant in the hippocampal tissues of TLE than in controls (Figure 6A). Correlation analysis (Figure 9D) showed only GLS and DLD of DECRGs were closely related to CD56dim natural killer cells, Effector memory CD4 T cells, Gamma delta T cells, and Monocyte.

**FIGURE 10:**
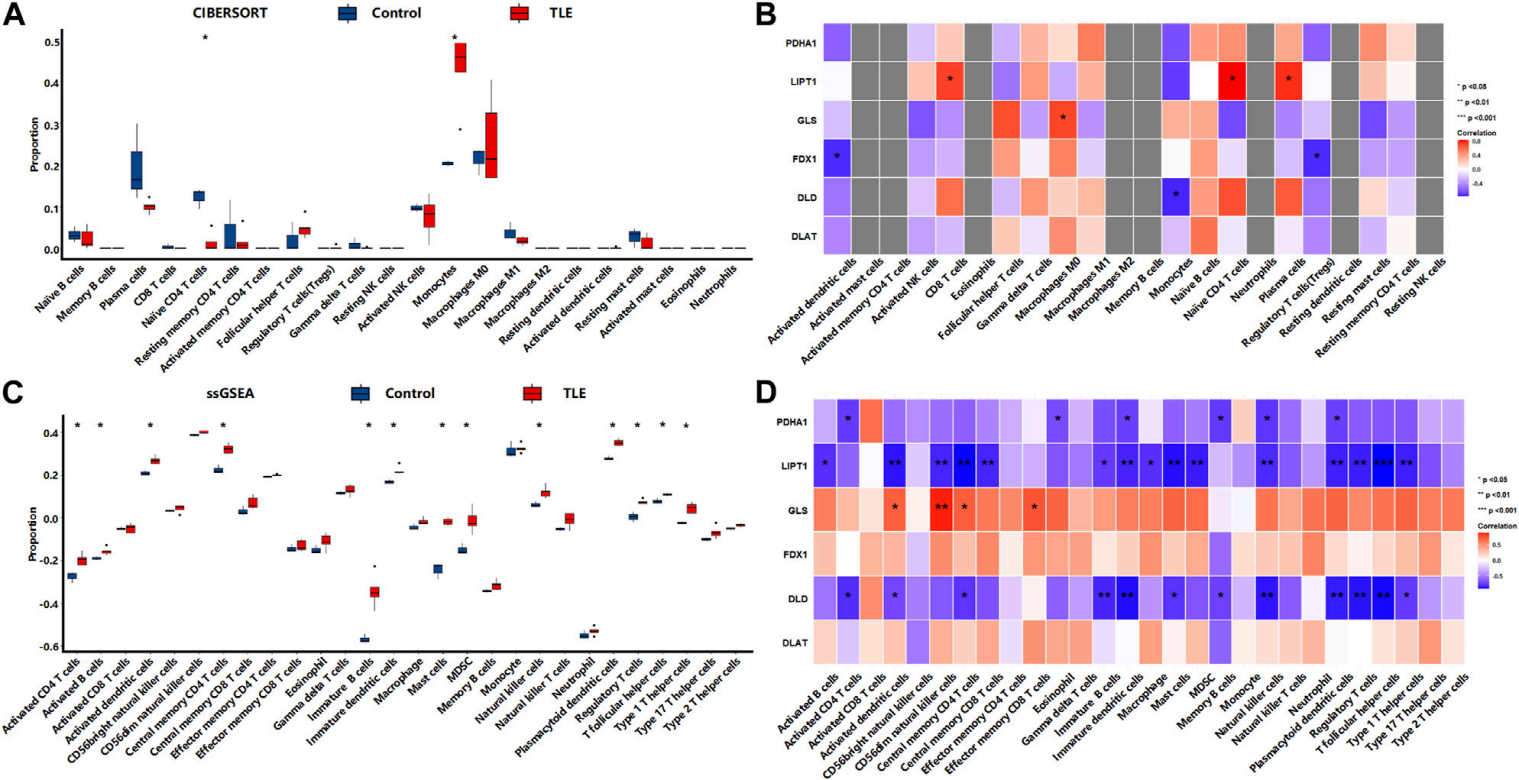
The immune cells infiltration analysis in the chronic stage of TLE. **(A,B)** The abundant of immune cell in the hippocampus of TLE of CIBERSORT algorithm and the correlation with DECRGs. **(C,D)** The abundant of immune cell in the hippocampus of TLE of ssGSEA algorithm and the correlation with DECRGs.

In the chronic stage, CIBERSORTx analysis (Figure 10A) showed that among 22 types of immune cells, Monocytes (*p* < 0.05) were more abundant in the hippocampal tissues of TLE than in controls (Figure 6A). Naïve CD4 T cells (*p* < 0.05) significantly decreased in TLE hippocampal samples compared to controls. Correlation analysis (Figure 10B) showed LIPT1 was related to the abundance of CD8 T cells, Naïve CD4 cells, and plasma cells; GLS was related to the abundance of Macrophages M0; FDX1 are closely related to the abundance of Activated dendritic cells and Regulatory T cells; DLD was related to the abundance of Monocytes. The ssGSEA analysis (Figure 10C) showed that among 28 types of immune cells, Activated CD4 T cells (*p* < 0.05), Activated B cells (*p* < 0.05), Activated dendritic cells (*p* < 0.05), Central memory CD4 T cells (*p* < 0.05), Effector memory CD4 T cells (*p* < 0.05), Immature B cells (*p* < 0.05), Immature dendritic cells (*p* < 0.05), Mast cells (*p* < 0.05), MDSC(*p* < 0.05), Natural killer cells (*p* < 0.05), Plasmacytoid dendritic cells (*p* < 0.05), Regulatory T cells (*p* < 0.05), T follicular helper cells (*p* < 0.05), and Type 1 T helper cells (*p* < 0.05) were more abundant in the hippocampal tissues of TLE than in controls (Figure 6A). Correlation analysis (Figure 10D) showed PDHA1 was closely related to the abundance of Activated CD4 T cells, Eosinophil, Immature dendritic cells, Memory B cells, Natural killer cells and Plasmacytoid dendritic cells; LIPT1 was closely related to the abundance of Activated B cells, Activated dendritic cells, CD56dim natural killer cells, Central memory CD4 T cells, Central memory CD4 T cells, Central memory CD8 T cells, Immature dendritic cells, Immature B cells, Macrophage, Mast cells, MDSC, Natural killer cells, Plasmacytoid dendritic cells, Regulatory T cells, T follicular helper cells, Type 1 T helper cells; GLS was related to the abundance of Activated dendritic cells, CD56dim natural killer cells, Central memory CD4 T cells, Effector memory CD8 T cells; DLD was related to the abundance of Activated CD4 T cells, Activated dendritic cells, Central memory CD4 T cells, Immature dendritic cells, Immature B cells, Mast cells, Memory B cells, Natural killer cells, Plasmacytoid dendritic cells, Regulatory T cells, T follicular helper cells, Type 1 T helper cells.

These results demonstrated that DECRGs were closely related to the abundance of immune cells in hippocampus tissues of TLE, especially macrophages and T cells, and the relationship between them was different in occurrent and progression of TLE: DECRGs were associated with the abundance of most infiltered immune cells in the acute phase of TLE, while the association significantly weakened in the latent phase; and in the chronic phase, DECRGs were associated with the abundance of many subclasses of T cells.

### Diagnostic values of DECRGs in identifying TLE

**FIGURE 11:**
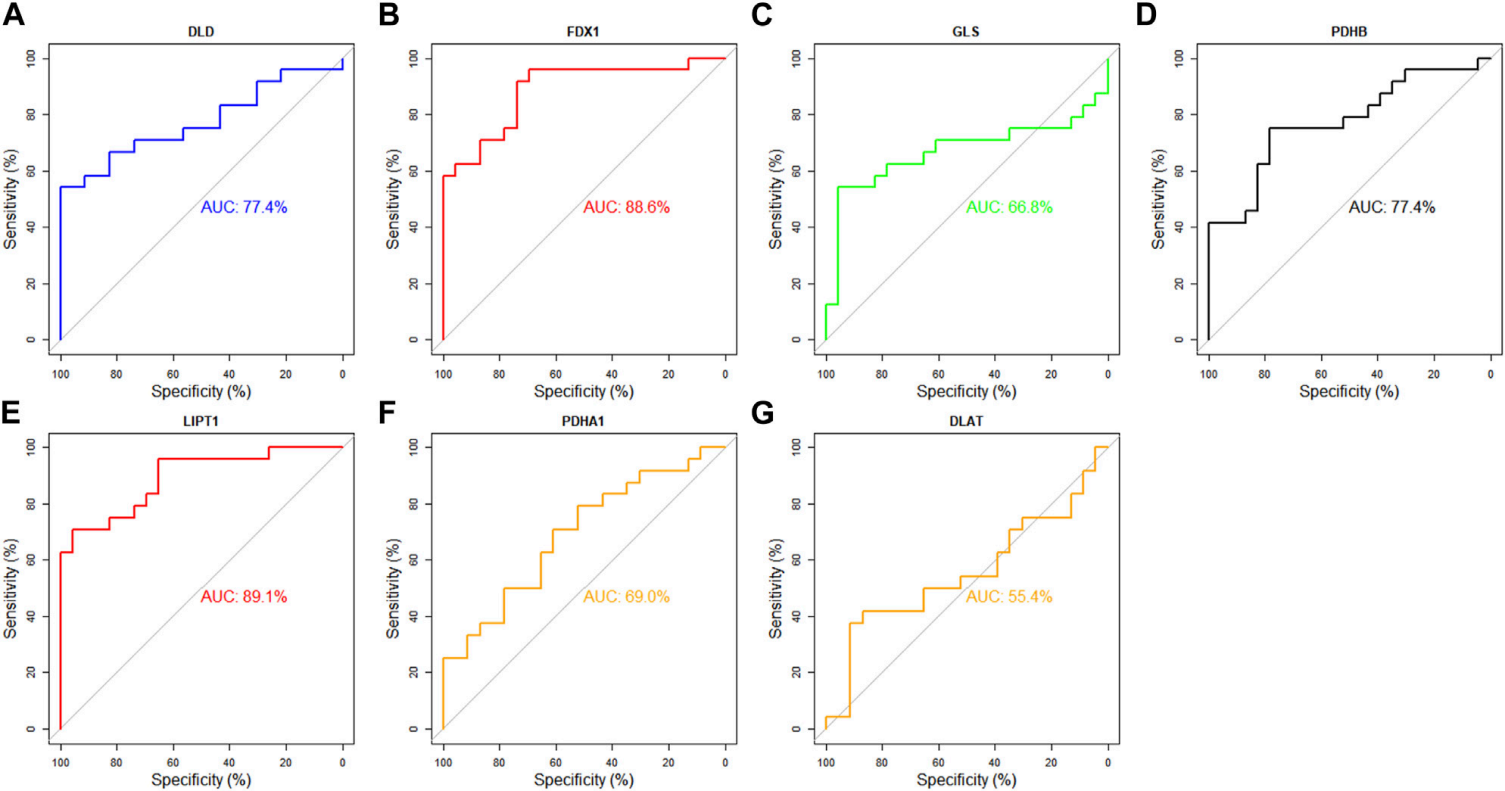
Analysis of the diagnostic value of DECRGs for TLE. The ROC curve of **(A)** DLD, **(B)** FDX1, **(C)** GLS, **(D)** PDHB, **(E)** LIPT1, **(F)** PDHA1, **(G)** DLAT.

Next, the diagnostic value of DECRGs for TLE was assessed using the ROC curve and AUC value in the E-MTAB-3123 data and Sample data. The AUC value of DECRGs in distinguish TLEs and controls were following (Figure 11): DLD (AUC = 77.4%), FDX1 (AUC = 88.6%), GLS (AUC = 66.8%), PDHB (AUC = 77.4%), LIPT1 (AUC = 89.1%), PDHA1 (AUC = 69%), and DLAT (AUC = 55.4%); Furthermore in the Sample data, the AUC value of DECRGs in distinguish TLEs and controls were following (Supplementary Figure S2): FDX1 (AUC = 100%), LIPT1 (AUC = 75%) DLD (AUC = 83.3%), GLS (AUC = 100%), PDHB (AUC = 83.3%), PDHA1 (AUC = 100%), and DLAT (AUC = 83.3%). These results suggested that LIPT1, FDX1, DLD, and PDHB could be the crucial genes in neuronal cuproptosis for TLE and have potential value for identifying TLE.

### The expression of LIPT1, FDX1, DLD, and PDHB

**FIGURE 12:**
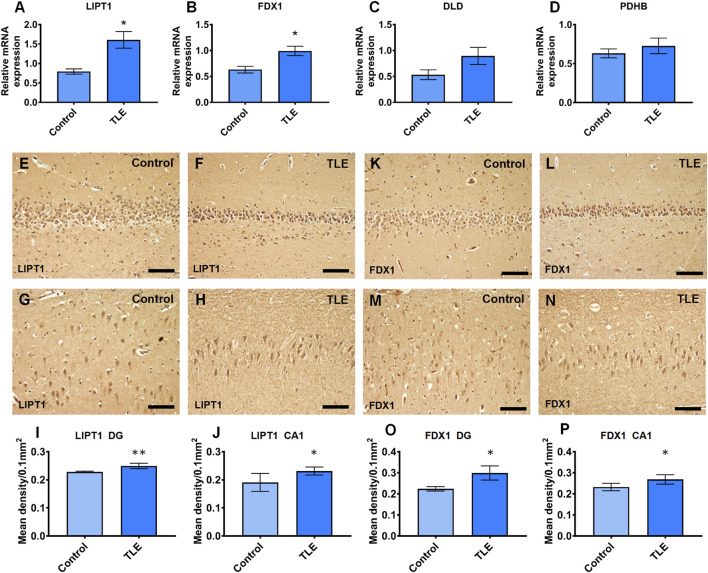
The real-time PCR validation of expression differently expressed cuproptosis-related genes which had the good diagnotic value of TLE. **(A)** The relative mRNA expression of LIPT1 (controls vs. TLEs *p* = 0.0359); **(B)** The relative mRNA expression of FDX1 (controls vs. TLEs *p* = 0.0384); **(C)** The relative mRNA expression of DLD (controls vs. TLEs *p* = 0.1667); **(D)** The relative mRNA expression of PDHB(controls vs. TLEs *p* = 0.5727); **(E–J)** The immunoreaction of LIPT1 in human hippocampus (controls vs. TLEs DG: *p* = 0.008; CA1: *p* = 0.0437); **(K–P)** The immunoreaction of FDX1 in human hippocampus (controls vs. TLEs DG: *p* = 0.0109; CA1: *p* = 0.0457).

To validate the mRNA expression of LIPT1, FDX1, DLD, and PDHB in the hippocampus of TLE, we performed real-time PCR experiments using human resected surgery specimens and control specimens (autopsy specimens). The results showed that LIPT1 and FDX1 expression significantly upregulated in the hippocampus of TLE compared to controls (Figures 12A, B, controls vs. TLEs *p* = 0.0359 and *p* = 0.0384, respectively). However, there were no differences in DLD and PDHB expression between the hippocampus of TLEs and controls (Figures 12C, D, controls vs. TLEs *p* = 0.1667 and *p* = 0.5727, respectively). Furthermore, the IHC results also showed the LIPT1 and FDX1 were significantly upregulated in the hippocampus DG and CA1 of TLE compared to controls (Figures 9E–P, LIPT1, controls vs. TLEs DG: *p* = 0.008, CA1: *p* = 0.0437; FDX1, controls vs. TLEs DG: *p* = 0.0109, CA1: *p* = 0.0457).

Therefore, according to the above results, we believed that LIPT1, FDX1, DLD, and PDHB are involved in the cuproptosis of hippocampal neurons of TLE patients, thus causing the infiltration of immune cells, leading to the occurrence and development of TLE (Supplementary Figure S3).

### Drugs targeting LIPT1, FDX1, DLD, and PDHB

Based on the above results, we explored the drugs targeting LIPT1, FDX1, DLD, and PDHB using Enrichr web, and found that valproic acid (VPA) targeted LIPT1, FDX1, DLD, and PDHB, and obtained the highest combined score, but the adjusted *p*-value was not significant (Table 2). Furthermore, chlorzoxazone might be a good drug for restricting the cuproptosis, since it targets LIPT1, FDX1, and DLD which are the key genes of cuproptosis according to a previous study (Table 2) (Tsvetkov et al., 2022). In addition, the piperlongumine targeting LIPT1 and PDHB might be another drug that inhibits cuproptosis (Table 2). However, these results need to be confirmed in further studies.

**TABLE 2:** Drugs Targeting LIPT1, FDX1, DLD, and PDHB from Enrichr database.

Drug name	*p*-value	Adjusted *p*-value	Combined score	Target genes
valproic acid CTD 00006977	0.0298	0.0637	164219.0132	LIPT1; FDX1; PDHB; DLD
flavin adenine dinucleotide TTD 00008045	0.0022	0.0467	4077.1879	DLD
brimonidine CTD 00000810	0.0022	0.0467	4077.1879	DLD
ferric ammonium citrate CTD 00000709	0.005	0.0467	1470.1975	PDHB
tonzonium bromide PC3 DOWN	0.0139	0.0547	411.4284	LIPT1
piperlongumine HL60 DOWN	0.0022	0.0467	309.921	LIPT1; PDHB
chlorzoxazone HL60 DOWN	0.001	0.0467	301.8211	LIPT1; FDX1; DLD
corticosterone	0.0355	0.0637	123.1709	FDX1
vitinoin CTD 00007069	0.0086	0.0467	117.342	PDHB; DLD
glibenclamide HL60 DOWN	0.0243	0.0637	52.1113	FDX1; DLD
lobeline HL60 DOWN	0.0308	0.0637	42.6513	LIPT1; DLD
clindamycin HL60 DOWN	0.0355	0.0637	37.7728	LIPT1; FDX1
cyclosporin A CTD 00007121	0.046	0.0709	29.0714	FDX1; PDHB; DLD

LIPT1, lipoyltransferase 1; DLD, dihydrolipoyl dehydrogenase; PDHB, pyruvate dehydrogenase E1 component subunit beta; FDX1, adrenodoxin.

## Discussion

In the study, we analyzed differentially expressed genes in six hippocampus samples which were removed from intractable TLE compared to autopsy controls (Sample dataset). The results were validated using the E-MTAB-3123 dataset in the ArrayExpress dataset. Four DECRGs (LIPT1, GLS, PDHA1, and CDKN2A) were identified in the Sample data. The E-MTAB-3123 dataset had seven DECRGs (LIPT1, DLD, FDX1, GLS, PDHB, PDHA1, and DLAT). However, only LIPT1 was consistently upregulated in both the Sample dataset and the E-MTAB-3123 dataset. In addition, these DECRGs are involved in the TCA cycle and pyruvate metabolism which are critical for cell cuproptosis (Tsvetkov et al., 2022). And DECRGs were associated with the abundance of macrophages and T cells in the hippocampus of TLE. Moreover, LIPT1, FDX1, DLD, and PDHB were related to the identification of TLE. Further, the real-time PCR and IHC confirmed that LIPT1 and FDX1 were upregulated in TLE compared to controls. And some drugs targeting LIPT1, FDX1, DLD, and PDHB were identified using the Enrichr online database. Thus, these results suggested that DECRGs are directly related to TLE, and LIPT1 and FDX1 could be key targets for inhibiting neuronal cuproptosis.

A recent study demonstrated that cuproptosis is a new regulated cell death (RCD) pattern which is dependent on the mitochondrial TCA cycle and differs from previous RCD forms, including apoptosis, ferroptosis, and necroptosis (Tsvetkov et al., 2022). As a novel RCD, the role and mechanism of cuproptosis in TLE remain unknown. In this study, four DECRGs in the sample data and seven out of ten key cuproptosis-related genes significantly upregulated in TLE compared to controls. Moreover, these DECRGs regulated the mitochondrial TCA cycle, pyruvate metabolism, and the cell cycle. Therefore, these cuproptosis-related genes may be involved in hippocampal neuronal loss during TLE. In addition, metallic copper nanoparticles have been confirmed to induce cell cycle arrest in the G2/M phase, mitochondrial membrane depolarization, DNA degradation, and chromosome condensation, resulting in cell death in melanoma cell lines (Lv et al., 2022). Furthermore, the knockout of cuproptosis-related genes, including LIPT1, FDX1, DLD, DLAT, PDHA1, LIAS, and PDHB, can rescue cell cuproptosis (Tsvetkov et al., 2022). In addition, copper directly induces cuproptosis by binding to components of the TCA cycle, not the electron transcript chain, leading to the shrinking of the spare capacity for mitochondrial respiration (Supplementary Figure S3) (Tsvetkov et al., 2022). Another concern is that the ketogenic diets suppress seizure through mitochondrial acetyl-CoA/TCA cycle metabolism (Alqahtani et al., 2020). Relevant to above point, mitochondrial metabolism abnormality is the critical pathogenesis associated with the development of epilepsy, and ketogenic diets affects the phenomena (Alqahtani et al., 2020). Furthermore, pyruvate administration significantly reduced the occurrence of seizures in patients with Leigh syndrome (Koga et al., 2012). Thus, we hypothesized that copper disrupted the TCA cycle and subsequent mitochondrial respiration, promoting neuronal death (cuproptosis), thus contributing to hippocampal neuronal loss in TLE. However, it is unclear whether these cuproptosis-related genes plays important roles in TLE.

LIPT1, which encodes lipoyltransferase 1, could be the key gene responsible for cuproptosis in TLE. It has been identified that LIPT1 was essential for the lipoate transferring to the E2 subunits of the 2-ketoacid dehydrogenases associated with the mitochondrial TCA cycle, and LIPT1 deficiency led to the impairment of the TCA cycle metabolism (Solmonson et al., 2022). LIPT1 has been shown to support lipogenesis and maintain metabolic homeostasis of oxidative and reductive glutamine (Ni et al., 2019). Apart from secondary pyruvate dehydrogenase complex deficiency and fatal lactic acidosis, LIPT1 deficiency was also the cause of Leigh disease (Soreze et al., 2013; Stowe et al., 2018; Solmonson et al., 2022). However, the role of LIPT1 in the epileptogenesis and progression of TLE is not well understood. Our results showed a higher level of LIPT1 expression in the hippocampus of TLE by analyzing the data from surgically removed samples and the ArrayExpress data. LIPT1 may be a good diagnostic indicator of TLE (AUC = 89.1% in E-MTAB-3123 data and AUC = 75% in Sample data, respectively). Therefore, we speculated that high levels of LIPT1 might promote seizures and the progression of TLE by disrupting TCA cycle in the mitochondria and subsequently triggering neuronal cuproptosis. LIPT1 has been reported to be associated with immune response signaling pathways, including interferon (IFN)γ and IFNα (Lv et al., 2022). Further PPI network analysis revealed that DECRGs were enriched in metabolism, cell cycle, and cell senescence pathways, suggesting that increased LIPT1 might inhibit hippocampal neuronal proliferation and promote neuronal death.

Cell death often releases pro-inflammatory factors, forming an inflammatory microenvironment to promote the epileptogenesis of TLE, which might promote peripheral immune cell infiltration into epileptic brain tissues (Zaben et al., 2021). Some studies have substantiated the effects of peripheral immune cells in the resected epileptic foci of pediatric and adult epilepsy patients; and in the infiltrated proinflammatory T cells, CD4^+^ T cells predominated (Gales and Prayson, 2017; Xu et al., 2018). Importantly, the CD4^+^ and CD8^+^ T cells infiltrating the human epileptic foci were positively related to seizure frequency, and the animal model also confirmed these results (Xu et al., 2018). A recent study showed the cell signatures of infiltrated Th1 and Th17 cells from drug-refractory epilepsy and demonstrated that the interaction between T cells and microglia promotes inflammation in TLE (Kumar et al., 2022). Additionally, in an electric kindling mouse model, the brain parenchyma was infiltrated by CD4^+^ and CD8^+^ T cells 24 h after a maximal seizure and reached a maximal value at 48 h but was undetectable on 7th day (Silverberg et al., 2010). Another study reported that CD4^+^ T cells were observed 96 h after status epilepticus (Vinet et al., 2016). Thus, T-cell infiltration varies widely depending on the timing of different studies (Hou et al., 2022). Further, in the plasma of patients with focal onset epilepsy, CD4 T+ cell populations were also found to be more abundant than in controls (Ouédraogo et al., 2021). A study also suggest that epilepsy is linked to a proinflammatory Th17/Th1 phenotype in CD4^+^ T cells (Ouédraogo et al., 2021). Further, IL-17 producing CD4^+^ T cells triggered neuronal death in a human iPSC model of Parkinson’s disease (Sommer et al., 2018). In our study, CD4^+^ T cell and Th17/Th1 phenotypes of CD4^+^ T cells were more abundant in the hippocampus of TLE patients than in controls, and we found that LIPT1, FDX1, DLD, and PDHB expression were positively correlated with the abundance of activated CD4^+^ T cells and Th1 CD4^+^ T cells, while Th17 CD4^+^ T cells showed the reverse relationship. Furthermore, DECRGs were associated with the abundance of most infiltered immune cells in the acute phase of TLE, while the association was significantly weakened in the latent phase, and in the chronic phase DECRGs were associated with the abundance of many subclasses of immune cells. These results suggested that DECRGs might influence neuronal cuproptosis in TLE by modulating the immune microenvironment and promoting the occurrence and development of TLEs.

To examine the potential value of DECRGs as diagnostic markers for TLE, we analyzed seven DECRGs using E-MTAB-3123 data and found that LIPT1, FDX1, DLD, and PDHB had high accuracy (AUC >0.7) in TLE prediction, supporting these genes as potential biomarkers or intervention targets for TLE. Moreover, LIPT1 upregulated the lipoylation of DLAT and DLST, and also enhanced PDHA1 phosphorylation and PDH activity which influenced pyruvate metabolism and the subsequent TCA cycle (Ni et al., 2019). In addition, human LIPT1 deficiency cause epilepsy, metabolic abnormalities, and developmental delay (Ni et al., 2019). Furthermore, FDX1 is essential in kickstarting the radical chain reaction of lipoly synthase (LIAS) and FDX1 knockout shows a strong lipoylation defect of the E2 subunits of DLAT and DLST of pyruvate dehydrogenase (PDH). In this study, LIPT1 and FDX1 were the valuable diagnostic indicator of TLE (AUC = 89.1% and AUC = 88.6% in E-MTAB-3123 dataset and AUC = 75% and AUC = 100% in Sample dataset respectively) and was related to the abundance of hippocampus-infiltrated immune cells. Next, we validated that LIPT1 and FDX1 were significantly upregulated in the hippocampus of TLE compared to controls using real-time PCR and immunohistochemical staining, but not DLD and PDHB. According to previous study, FDX1 and LIPT1 independent knockout can rescue cells from copper toxicity (Tsvetkov et al., 2022). Thus, LIPT1 and FDX1 may be the key gene responsible for neuronal cuproptosis in TLE (see Supplementary Figure S3). Thus, targeting LIPT1 and FDX1 may rescue hippocampal neuronal cuproptosis in TLE.

Valproic acid (VPA), a first-line antiseizure drug, confers a neuroprotective environment after brain injury by modulating brain tissue metabolism (Bhatti et al., 2021). In this study, we found that VPA inhibited LIPT1, FDX1, DLD, and PDHB and had the highest score, although the adjusted *p*-value was not significant. Unfortunately, hippocampal neurogenesis is suppressed by VPA, resulting in cognitive deficits (Romoli et al., 2019). Thus, VPA might not be a good choice to inhibit neuronal cuproptosis in the hippocampus in TLE. However, chlorzoxazone, a K_Ca_ channel activator, displays potential antiseizure effects by regulating neuronal excitability (Kobayashi et al., 2008). Moreover, chlorzoxazone reduces hippocampal neuronal death and the inflammatory response to rescue cognitive deficits in Alzheimer’s disease (Bai and Ma, 2020). In addition, the results of the study showed that chlorzoxazone inhibited LIPT1, FDX1, and DLD expression, indicating that chlorzoxazone could inhibit hippocampal neuronal cuproptosis in TLE. In addition, our results revealed that piperlongumine might suppress hippocampal neuronal cuproptosis in TLE by inhibiting the expression of LIPT1 and PDHB. It has been reported that piperlongumine improves cognitive impairment by increasing hippocampus neurogenesis (Go et al., 2018). Piperlongumine also exerts biological effects by suppressing NF-κ B activity and assembly of the NLRP3 inflammasome (Kim et al., 2018; Shi et al., 2021). Thus, chlorzoxazone and piperlongumine may be drugs that target cuproptosis to improve the seizure and progression of TLE. However, these results need to be confirmed through further research.

## Conclusion

In summary, cuproptosis may be a potential target for seizure control, and cuproptosis-related gene signatures may be new predictors for the diagnosis of TLE, which provides new clues for exploring the role of cuproptosis in TLE. Sincerely, more research is needed to further confirm these results to increase the knowledge of TLE for preventing and controlling seizures.

## Limitation

Our study had some limitations. First, hippocampal specimens from age-matched controls were sparse, although the analyzed results of screened genes in our resected samples dataset and the E-MTAB-3123 dataset suggested, to a certain extent, that four genes (LIPT1, FDX1, DLD, and PDHB) were good identifiable indicators, and targeting these genes could repress hippocampal neuronal cuproptosis to improve the seizure and progression of TLE. However, no data from a large sample size (*n* > 50) or clinical information for TLEs and controls were available for further validation of our results. Further studies are required to address these questions. Second, although DECRGs were found to be involved in the immune infiltration of epileptic foci, whether the LIPT1 and FDX1 are the key genes is unknown. Future research should be conducted to confirm these results using biological experiments. Third, we should recognize that the expressive forms of the FDX1, DLD, and PDHB genes were not consistent in the E-MTAB-3123 data and our resected sample data, although real-time PCR and IHC were used to validate the expression of these genes. Therefore, the signatures of LIPT1, FDX1, DLD, and PDHB need to be further validated by exploiting clinical samples or other data from public databases, and biological evidence is needed in addition to the statistical results in our study.

## Data Availability

The data presented in the study are deposited in the Array Express and Gene Expression Omnibus database repository, accession number E-MTAB-3123, E-MTAB-13092, GSE49030, GSE88992, GSE49849 and GSE14763 (https://www.ebi.ac.uk/biostudies/arrayexpress and https://www.ncbi.nlm.nih.gov/geo/).

## Supplementary material

SUPPLEMENTARY FIGURE S1 The copper ion concentration and the expression level of ceruloplasmin in the hippocampus of TLEs and controls. **(A)** The Cu concentration in the hippocampus of TLEs and controls (controls vs TLEs P=0.0547); **(B)** The expression level of ceruloplasmin in the hippocampus of TLEs and controls in Sample dataset (controls vs TLEs P=0.3736) and E-MTAB-3123 dataset (controls vs TLEs P=0.5575) respectively.

SUPPLEMENTARY FIGURE S2 The analysis of the diagnostic value of DECRGs for TLE. **(A)** The ROC curve of DLD; **(B)** The ROC curve of FDX1; **(C)** The ROC curve of GLS; **(D)** The ROC curve of PDHB; **(E)** The ROC curve of LIPT1; **(F)** The ROC curve of PDHA1; **(G)** The ROC curve of DLAT.

SUPPLEMENTARY FIGURE S3 Schematic of cuproptosis signaling pathway.

SUPPLEMENTARY TABLE S1 The difference of key cuproptosis related genes between TLEs and controls in sample data.

SUPPLEMENTARY TABLE S2 The difference of key cuproptosis related genes between TLEs and controls in E-MTAB-3123 data.
