# Supplementary figures and images for: Cuproptosis-related genes signature and validation of differential expression and the potential targeting drugs in temporal lobe epilepsy
Source: Front Pharmacol. 2023 Jun 26;14:1033859. doi: 10.3389/fphar.2023.1033859 (PMC10330702; doi:10.3389/fphar.2023.1033859)

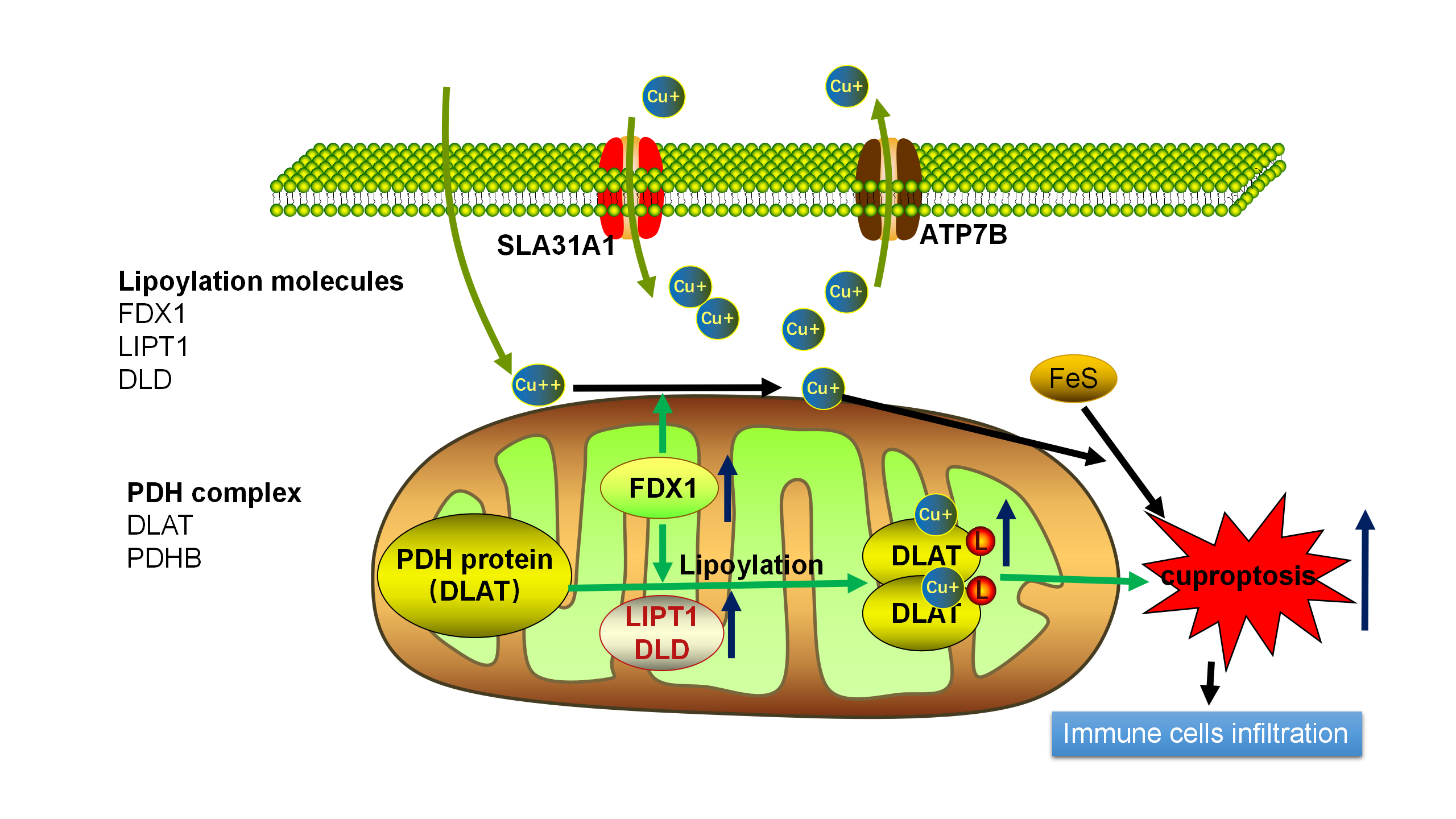

Supplement: Supplementary file 1 [file Image3.tif]

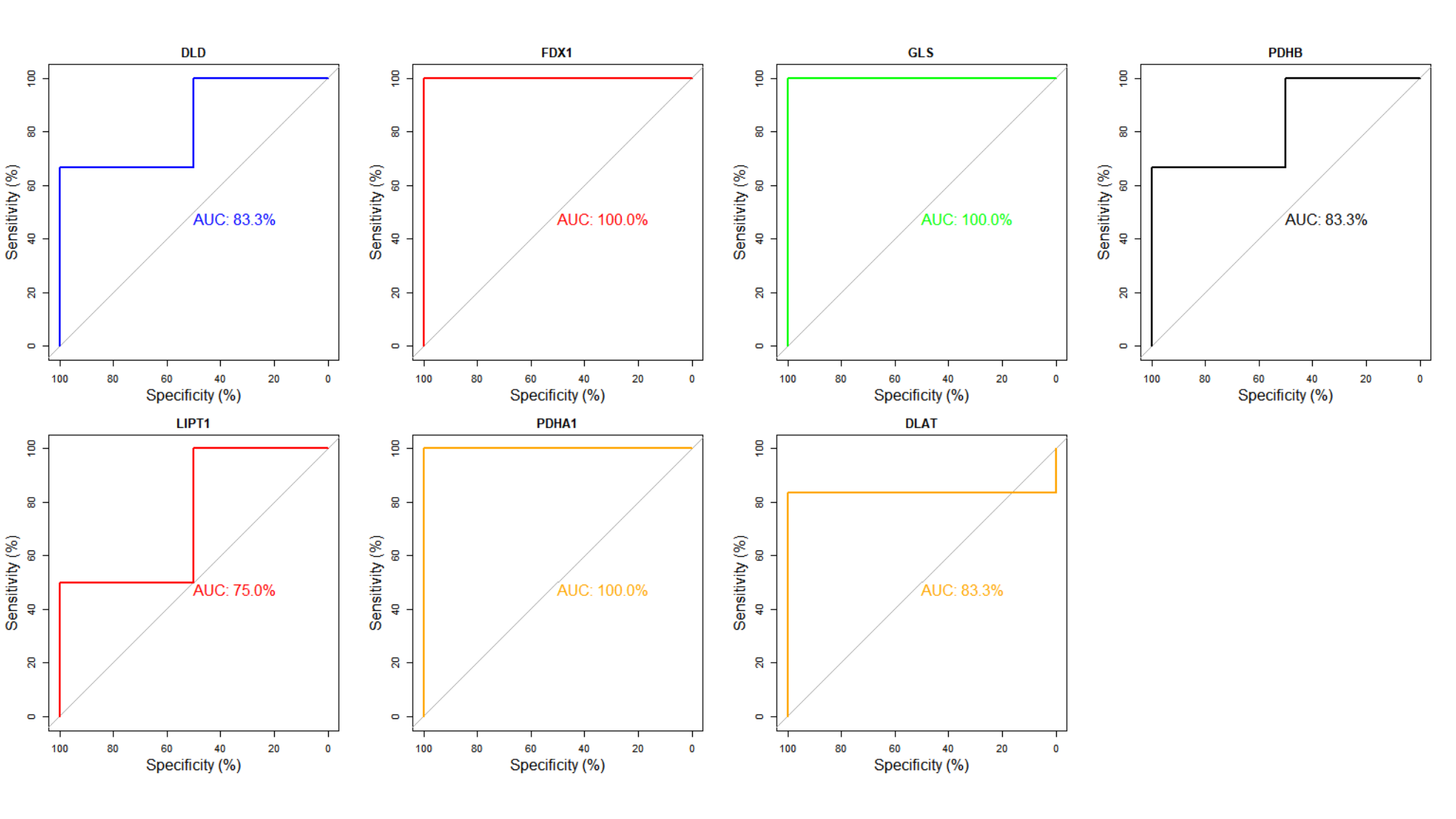

Supplement: Supplementary file 2 [file Image2.tif]

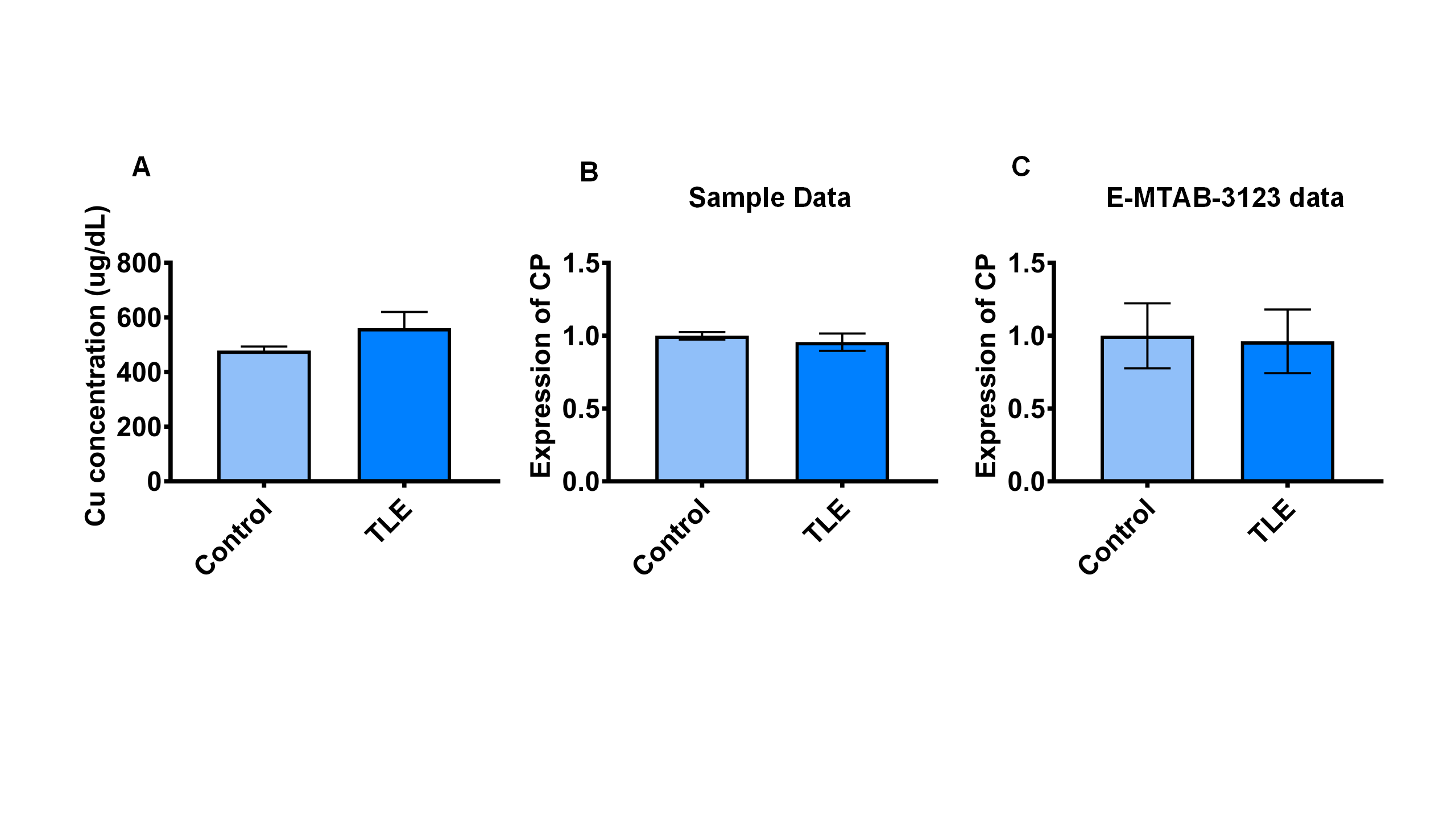

Supplement: Supplementary file 3 [file Image1.tif]
