# Supplementary material for: Cuproptosis-related genes signature and validation of differential expression and the potential targeting drugs in temporal lobe epilepsy
Source: Front Pharmacol. 2023 Jun 26;14:1033859. doi: 10.3389/fphar.2023.1033859 (PMC10330702; doi:10.3389/fphar.2023.1033859)
Supplement: Supplementary file 4 [file DataSheet1.DOCX]

Supplementary Table 1. The difference of key cuproptosis related genes between TLEs and controls in Sample data

| **Sample data** | | | |
| --- | --- | --- | --- |
| Genes | log2FC | Pvalue | EP VS Control |
| LIPT1 | 1.34 | 7.04E-03 | UP |
| CDKN2A | -1.94 | 4.38E-05 | Down |
| PDHA1 | -1.06 | 3.01E-03 | Down |
| GLS | -1.43 | 1.04E-02 | Down |
| SLC31A1 | -0.85 | 2.34E-03 | NOT |
| ATP7B | -0.61 | 8.30E-02 | NOT |
| DLD | -0.46 | 1.48E-01 | NOT |
| DLAT | -0.45 | 1.69E-01 | NOT |
| MTF1 | 0.34 | 2.07E-01 | NOT |
| LIAS | 0.40 | 2.36E-01 | NOT |
| FDX1 | -0.22 | 2.96E-01 | NOT |
| PDHB | 0.12 | 5.87E-01 | NOT |

Supplementary Table 2. The difference of key cuproptosis related genes between TLEs and controls in E-MTAB-3123 data

| **E-MTAB-3123** | | | |
| --- | --- | --- | --- |
| Genes | logFC | PValue | EP VS Control |
| DLD | 0.74 | 1.60E-08 | UP |
| FDX1 | 0.37 | 1.97E-07 | UP |
| GLS | 0.51 | 7.48E-07 | UP |
| PDHB | 0.48 | 8.45E-07 | UP |
| LIPT1 | 0.57 | 2.41E-05 | UP |
| PDHA1 | 0.22 | 7.10E-03 | UP |
| DLAT | 0.18 | 1.79E-02 | UP |
| SLC31A1 | 0.09 | 1.52E-01 | NOT |
| MTF1 | -0.11 | 5.82E-01 | NOT |
| LIAS | 0.05 | 6.48E-01 | NOT |
| CDKN2A | 0.07 | 7.43E-01 | NOT |
| ATP7B | 0.05 | 6.74E-02 | NOT |
